# Inhibition of IL-1R1/MyD88 signalling promotes mesenchymal stem cell-driven tissue regeneration

**DOI:** 10.1038/ncomms11051

**Published:** 2016-03-22

**Authors:** Mikaël M. Martino, Kenta Maruyama, Gisela A. Kuhn, Takashi Satoh, Osamu Takeuchi, Ralph Müller, Shizuo Akira

**Affiliations:** 1 WPI Immunology Frontier Research Center, Osaka University, 3-1 Yamada-oka, Suita, Osaka 565-0871, Japan; 2 Institute for Biomechanics, Leopold-Ruzicka-Weg 4, ETH Zurich, Zurich 8093, Switzerland; 3 Institute for Virus Research, Kyoto University, 53 Shogoin Kawara-cho, Sakyo-ku, Kyoto 606-8507, Japan

## Abstract

Tissue injury and the healing response lead to the release of endogenous danger signals including Toll-like receptor (TLR) and interleukin-1 receptor, type 1 (IL-1R1) ligands, which modulate the immune microenvironment. Because TLRs and IL-1R1 have been shown to influence the repair process of various tissues, we explored their role during bone regeneration, seeking to design regenerative strategies integrating a control of their signalling. Here we show that IL-1R1/MyD88 signalling negatively regulates bone regeneration, in the mouse. Furthermore, IL-1β which is released at the bone injury site, inhibits the regenerative capacities of mesenchymal stem cells (MSCs). Mechanistically, IL-1R1/MyD88 signalling impairs MSC proliferation, migration and differentiation by inhibiting the Akt/GSK-3β/β-catenin pathway. Lastly, as a proof of concept, we engineer a MSC delivery system integrating inhibitors of IL-1R1/MyD88 signalling. Using this strategy, we considerably improve MSC-based bone regeneration in the mouse, demonstrating that this approach may be useful in regenerative medicine applications.

## Abstract (web summary)

TLR and IL-1R1 ligands are danger signals released following tissue injury and during the healing response. Here, the authors show that IL-1β signalling via IL-1R1/MyD88 inhibits the Akt/GSK-3β/β-catenin pathway in mesenchymal stem cells, which suppresses their mobilization, proliferation, and differentiation into osteoblasts, processes necessary for bone regeneration.

## Graphical abstract

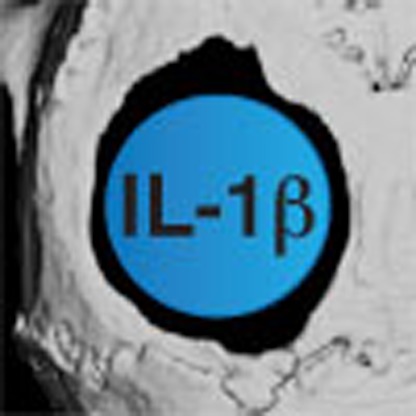

Although, the advancement of regenerative medicine will play a vital role in meeting the future healthcare challenges, the promises of regenerative therapies remain largely unrealized. For designing effective regenerative medicine strategies, we should better understand the interactions between the multiple actors that shape a regenerative environment. In particular, tissue injury is generally associated with an immune response, which is most likely a key regulator of the healing process^1^,^2^. Hence, in-depth understanding of the role of the immune system during tissue repair and regeneration could provide clues to therapeutic avenues for restoring damaged tissues, and controlling the immune regulations of tissue healing may become an attractive option in regenerative medicine^1^,^2^.

Unlike most tissues, bone possesses an innate capacity to regenerate following injury. The majority of bony injuries, when properly treated by re-apposition, heal without a permanent lesion. However, many clinical indications remain that require therapeutic intervention to augment bone regeneration such as large craniomaxillofacial defects, bone degeneration in patients with osteonecrosis, distal tibial fractures and periodontal disease^3^,^4^. Autologous bone grafting is currently the gold standard, but this approach is associated with numerous drawbacks, including donor-site morbidity, the availability of limited grafting material and compromised bone quality in patients with osteoporosis^5^. Therefore, extensive efforts have been made to develop bone regenerative strategies using various combinations of cells^4^, growth factors^6^ and biomaterials^7^. However, only few of these strategies have translated into clinical practice and none of them have become a standard in regenerative medicine. Efficacy, safety, practical, cost-effectiveness and regulatory issues often prevent the widespread therapeutic use of bone regenerative therapies^4^,^8^. In addition, one of the major challenges lies in the limited understanding of the cellular and molecular mechanisms that should be targeted to promote bone regeneration. Especially, understanding and subsequently controlling the immune regulations of bone regeneration could be crucial to improve the effectiveness of bone regenerative therapies^1^,^2^,^9^.

Commonly, tissue injury and the healing response lead to the release of various endogenous danger signals including Toll-like receptor (TLR) and interleukin-1 receptor, type 1 (IL-1R1) ligands^10^,^11^, which modulate the immune microenvironment. These danger signals are involved in the recruitment and the activation of immune cells engaged in host defence^11^,^12^. In addition, TLRs and IL-1R1 have been shown to influence the repair process of several tissues^13^,^14^,^15^,^16^,^17^,^18^,^19^,^20^,^21^,^22^,^23^. For example, the injury promoting effects of TLR4 is apparent in many organs, as seen by the protection of TLR4-mutant or -deficient mice after hepatic, renal, cardiac and cerebral ischemia reperfusion^13^,^14^,^15^,^16^,^19^. Similarly, IL-1R1 signalling critically regulates infarct healing^17^ and disruption of IL-1 signalling can improve the quality of wound healing^18^,^21^.

In this study, we explore the role of TLRs and IL-1R1 during bone regeneration, seeking to design regenerative strategies integrating a control of their signalling. We show that IL-1R1 signalling via the adaptor protein MyD88 negatively regulates bone regeneration, in the mouse. IL-1β is released at the bone injury site and inhibits the regenerative capacities of mesenchymal stem cells (MSCs). Mechanistically, IL-1R1/MyD88 signalling impairs MSC migration, proliferation and differentiation into osteoblasts, by inhibiting the Akt/GSK-3β/β-catenin pathway. Furthermore, we propose a MSC delivery system integrating inhibitors of IL-1R1/MyD88 signalling. Using this approach, we significantly improve MSC-based bone regeneration in a mouse critical size calvarial defect model, demonstrating that this approach may be useful in regenerative medicine applications.

## Results

### IL-1R1/MyD88 signalling negatively regulates bone regeneration

**Figure 1:**
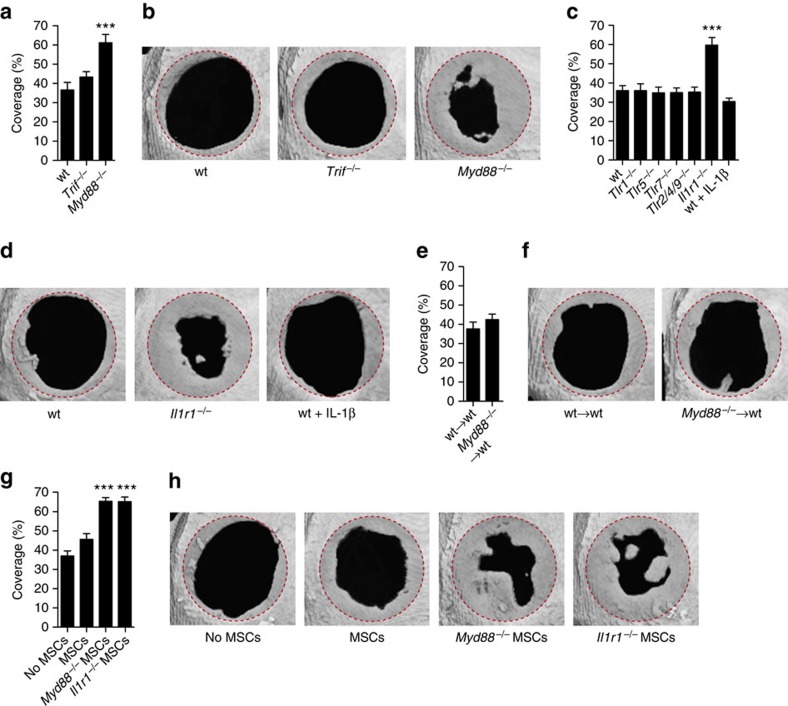
IL-1R1/MyD88 signalling negatively regulates MSC-driven bone regeneration. Critical size calvarial defects (5 mm diameter) in mice were treated with a fibrin matrix. Four weeks after treatment, bone regeneration was measured by microCT as coverage of the defect. (**a**,**b**) Regeneration in wt, *Myd88*^−/−^ and *Trif*^−/−^ mice. (**c**,**d**) Regeneration in wt, *Tlr1*^−/−^, *Tlr5*^−/−^, *Tlr7*^−/−^, *Tlr2/4/9*^−/−^ (triple knockout), *Il1r1*^−/−^ and in wt mice where 10 ng of IL-1β was co-delivered. (**e**,**f**) Regeneration in irradiated wt mice having received a bone marrow transplant from wt or *Myd88*^−*/*−^ mice. (**g**,**h**) Regeneration in wt mice induced by syngeneic wt, *Myd88*^−/−^ or *Il1r1*^−/−^ MSCs delivered in fibrin matrix. For **a**,**c**,**e** and **g**, data are means±s.e.m. (*n*≥6 per condition). ANOVA with Bonferroni *post hoc* test for pair-wise comparisons; ***P<0.001. Representative calvarial reconstructions are shown in **b**, **d**,**f** and **h**. Original defect area is shaded with a red dotted outline. ANOVA, analysis of variance.

To evaluate the role of TLRs and IL-1R1 during bone regeneration, we first analysed regeneration in mice deficient for MyD88 and TRIF, which are key adaptor proteins involved in TLR and IL-1R1 signalling transduction^12^. Among the available orthotopic models used for evaluating bone regeneration, we chose the critical size calvarial defect, because this model have gained a widespread reputation and use in the published literature, reporting valuable data from basic and applied research^24^. Moreover, the critical size calvarial defect model is very reliable and displays low variability^25^, making it ideal to compare bone regeneration in multiple knockout mice. Critical size calvarial bone defects (5 mm diameter) were created and filled with a standard fibrin matrix. The fibrin matrix was used to ensure clot formation in the defect and to standardize the model^8^, while the procedure did not significantly influence bone regeneration (Supplementary Fig. 1). Remarkably, 4 weeks post surgery, *Myd88*^−/−^ mice displayed a faster regeneration characterized by a better coverage of the defect with mineralized bone (Fig. 1a,b), suggesting that TLR1, TLR2, TLR4–9, and/or IL-1R1 influence bone regeneration, since their signalling depends on MyD88 (ref. 12). When bone regeneration was analysed in *Tlr1*^−/−^, *Tlr2/4/9*^−/−^ (triple knockout), *Tlr5*^−/−^, *Tlr7*^−/−^ and *Il1r1*^−/−^ mice, only *Il1r1*^−*/*−^ mice displayed better healing (Fig. 1c,d), indicating that IL-1R1/MyD88 signalling negatively regulates bone regeneration.

### IL-1R1/MyD88 signalling inhibits MSC-driven bone regeneration

Since IL-1R1/MyD88 signalling is well known for inducing mobilization and activation of immune cells^26^, we determined whether this signalling inhibits bone regeneration by acting on the immune cells recruited after injury and throughout the regeneration process. As a model, we reconstituted lethally irradiated wild-type (wt) mice by intravenously injecting bone marrow cells from *Myd88*^−/−^ mice, to have chimeric mice with bone marrow-derived immune cells unable to transduce IL-1R1/MyD88 signalling (Supplementary Fig. 2). While very few *Myd88*^−/−^ bone marrow MSCs could have been transferred in wt mice (0.001–0.01% of injected cells), *Myd88*^−/−^ MSCs most likely did not populate the bone marrow of irradiated wt mice, since the vast majority of MSCs does not go beyond lung and liver following intravenous injection^27^,^28^. Interestingly, *Myd88*^−/−^ chimeric mice did not display better healing (Fig. 1e,f), suggesting that IL-1R1/MyD88 signalling negatively regulates bone regeneration by acting on tissue-resident cells. Since osteoclasts derive from bone marrow monocytes, they were lacking MyD88 in *Myd88*^−/−^ chimeric mice. Therefore, we hypothesized that IL-1R1/MyD88 signalling negatively regulates bone regeneration by acting on osteoblasts and/or skeletal stem/progenitor cells including MSCs^29^,^30^,^31^,^32^,^33^.

To test whether IL-1R1/MyD88 signalling influences MSC-driven bone regeneration, we delivered syngeneic MSCs derived from bone of wt, *Myd88*^−/−^ or *Il1r1*^−/−^ mice into calvarial defects of wt mice, and we analysed regeneration after 4 weeks. MSCs from five isolates were pulled together and expended for three passages (Supplementary Fig. 3), before being delivered in bone defects through a fibrin matrix. Delivering wt MSCs resulted into a very slight improvement of regeneration compared with treatment without cells. In contrast, delivering *Myd88*^−/−^ or *Il1r1*^−/−^ MSCs induced significantly better regeneration (Fig. 1g,h). To check if the regeneration stimulated by *Myd88*^−*/*−^ and *Il1r1*^−*/*−^ MSCs relied on a better intrinsic capacity of these cells to differentiate towards osteoblasts or to proliferate, we compared them with wt MSCs *in vitro*. Without IL-1R1 ligands added to the cell culture medium, *Myd88*^−/−^, *Il1r1*^−/−^ and wt MSCs showed equal ability to differentiate and to proliferate (Supplementary Fig. 4). Therefore, we hypothesized that the better regeneration induced by *Myd88*^−/−^ and *Il1r1*^−/−^ MSCs was due to their inability to sense IL-1R1 ligands present at the injury site.

### IL-1β signalling via IL-1R1/MyD88 inhibits MSC functions

**Figure 2:**
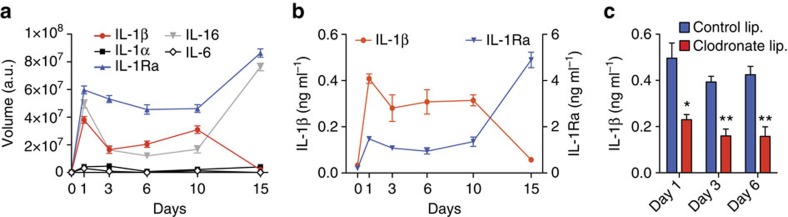
IL-1β is present at a significant concentration in bone defects within the first 10 days following injury. (**a**–**c**) Calvarial defects (4 mm diameter) were treated with fibrin matrices. Fibrin matrices and bone tissue surrounding the defects (1 mm farther) were collected at different time points. Interleukins present in the harvested tissues and in fibrin matrices were detected using an antibody array (**a**) and by enzyme-linked immunosorbent assay (**b**,**c**). (**c**) IL-1β concentrations found in mice that have been treated with control or clodronate liposomes (lip.). For panels **a**–**c**, data are means±s.e.m. (*n*≥3 per time point). *P<0.05, **P<0.01; Student's *t*-test.

Using a cytokine array, we profiled the cytokines present in the bone defect microenvironment during the first 2 weeks following injury (Supplementary Fig. 5). Importantly, among IL-1R1 ligands^26^, IL-1β as well as IL-1 receptor antagonist (IL-1Ra) were present at relatively high concentration within the first 10 days (Fig. 2a,b). After 10 days, IL-1β concentration starts to decline, while IL-1Ra concentration drastically increases afterwards, suggesting that the IL-1R1/MyD88 signalling occurs for at least 1 week post injury. Moreover, to gain insights about the cell types releasing IL-1β, we quantified the concentration of the cytokine in calvarial defects of mice where monocytes/macrophages were depleted by clodronate liposomes^34^, since these cells are known to release a large amount of IL-1β (ref. 26). In mice depleted of monocytes/macrophages (Supplementary Fig. 6), the concentration of IL-1β was about twice lower during the first 6 days following injury (Fig. 2c), demonstrating that monocytes/macrophages significantly contribute to the presence of IL-1β.

**Figure 3:**
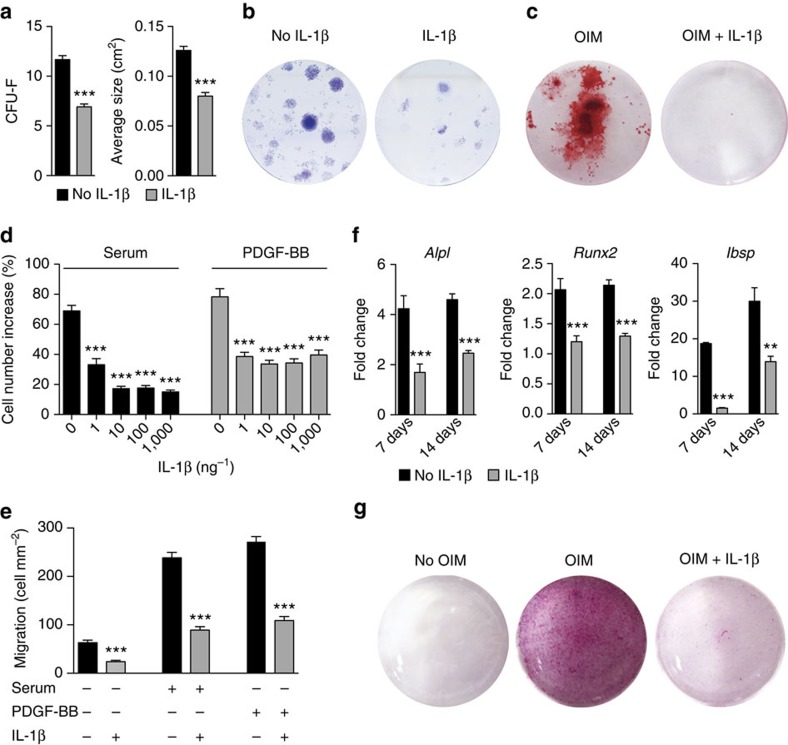
IL-1β inhibits c.f.u.-F proliferation migration and osteoblastic differentiation of MSCs. (**a**,**b**) Primary MSCs were seeded with or without IL-1β (1 ng ml^−1^ during 6 days). Graphs show c.f.u.-F and average size of colonies. Data are means±s.e.m. (*n=*6 independent isolates). Representative wells (9 cm^2^) are shown in **b**. (**c**) Primary MSCs were seeded in osteogenesis induction medium (OIM) for 28 days with or without IL-1β (1 ng ml^−1^ during 6 days). After 28 days, matrix mineralization was revealed with alizarin red staining. Representative wells are shown (2 cm^2^). (**d**) MSC proliferation was stimulated with 10% serum or PDGF-BB (5 ng ml^−1^), in the presence of IL-1β at increasing concentration. After 72 h, cell number increase was measured. (**e**) Migration of MSCs through a transwell was induced by 10% serum or by PDGF-BB (5 ng ml^−1^), in the presence of IL-1β (1 ng ml^−1^). After 6 h, the number of cells per square millimetre that passed through the transwell was counted. (**f**) MSCs in OIM were treated once with IL-1β (1 ng ml^−1^ during 4 days). After 7 and 14 days, expression of osteoblast-specific genes was determined by quantitative PCR. Fold changes in gene expression relative to MSCs cultured in normal medium are shown. *Alpl*, alkaline phosphatase; *Runx2*, runt-related transcription factor 2; *Ibsp,* integrin-binding sialoprotein. For **d**–**f**, data are means±s.e.m. (*n*≥3). **P<0.01, ***P<0.001; Student's *t*-test. (**g**) MSCs in OIM were treated once with IL-1β (1 ng ml^−1^) during 4 days. After 28 days, matrix mineralization was revealed with alizarin red staining. Representative wells (2 cm^2^) are shown.

**Figure 4:**
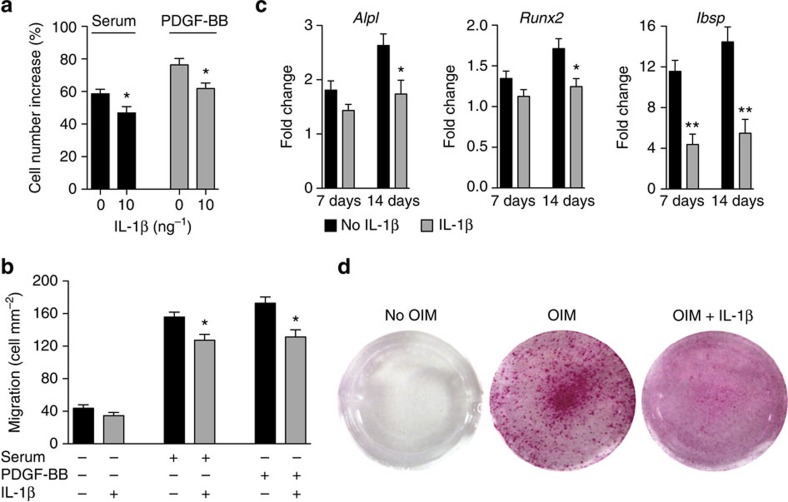
IL-1β slightly inhibits proliferation migration and differentiation of osteoblasts. (**a**) Osteoblast proliferation was stimulated with 10% serum or PDGF-BB (10 ng ml^−1^), in the presence of 5 ng ml^−1^ of IL-1β. After 72 h, the cell number increase was measured. Data are means±s.e.m. (*n*=3). (**b**). Osteoblast migration through a transwell was induced by 10% serum or PDGF-BB (5 ng ml^−1^), in the presence of IL-1β (1 ng ml^−1^). After 6 h, the number of cells per square millimetre that passed through the transwell was measured. Data are means±s.e.m. (*n*=3). (**c**) Osteoblasts in osteogenesis induction medium (OIM) were treated once with IL-1β (1 ng ml^−1^ during 4 days). (**d**) After 28 days, matrix mineralization was revealed with alizarin red staining. Representative wells are shown. For **a**–**c**, data are means±s.e.m. (*n*≥3). *P<0.05, **P<0.01; Student's *t*-test.

Next, we explored the impact of IL-1R1/MyD88 signalling on the regenerative capacities of MSCs. We focused on colony formation, proliferation, migration, differentiation and trophic function (for example, secretion of growth factors), considering that these functions are critical for the regenerative potential of MSCs. When primary (uncultured) bone-derived MSCs were seeded in the presence of IL-1β (1 ng ml^−1^), fibroblast colony-forming units (c.f.u.-F) and the average size of colonies were significantly lower (Fig. 3a,b; Supplementary Fig. 7a–c). In addition, IL-1β (1 ng ml^−1^) drastically inhibited the differentiation of the colonies towards osteoblasts (Fig. 3c; Supplementary Fig. 7d). Then, we analysed the effect of IL-1β on proliferation, migration, differentiation and trophic function, of bone-derived MSCs that have been expanded for three passages. Secretion of trophic factors from MSCs was not affected by IL-1β (Supplementary Fig. 8). Yet, IL-1β considerably inhibited MSC proliferation, when cells were co-stimulated with serum or with platelet-derived growth factor-BB (PDGF-BB), which is potent proliferation and migration factor for MSCs^35^ (Fig. 3d; Supplementary Fig. 9a). Similarly, transwell migration of MSCs towards serum or PDGF-BB was inhibited by IL-1β (Fig. 3e; Supplementary Fig. 9b). Regarding MSC differentiation, a single stimulation with a low concentration of IL-1β (1 ng ml^−1^) severely inhibited their differentiation into osteoblasts (Fig. 3f,g; Supplementary Fig. 9c) and into chondrocytes (Supplementary Fig. 10). In addition, proliferation, migration and osteoblastic differentiation of MSCs lacking IL-1R1 and/or MyD88 were not affected by IL-1β (Supplementary Fig. 11), confirming that the IL-1R1/MyD88 signalling pathway is responsible for the inhibitory effects of the cytokine. Notably, IL-1β inhibited colony formation, proliferation, migration and osteoblastic differentiation of bone marrow-derived MSCs (Supplementary Fig. 12). The inhibitory effects of IL-1β and their magnitudes were very similar to the ones observed with bone-derived MSCs. Therefore, we chose to use bone-derived MSCs for all the subsequent experiments involving MSCs. Lastly, we tested the effects of IL-1R1/MyD88 signalling on osteoblasts. IL-1β inhibited osteoblast proliferation, migration and differentiation (Fig. 4). However, the effects of the cytokine were less strong on osteoblasts compared with MSCs, suggesting that the inhibitory effects of IL-1R1/MyD88 signalling are more severe on progenitor cells.

**Figure 5:**
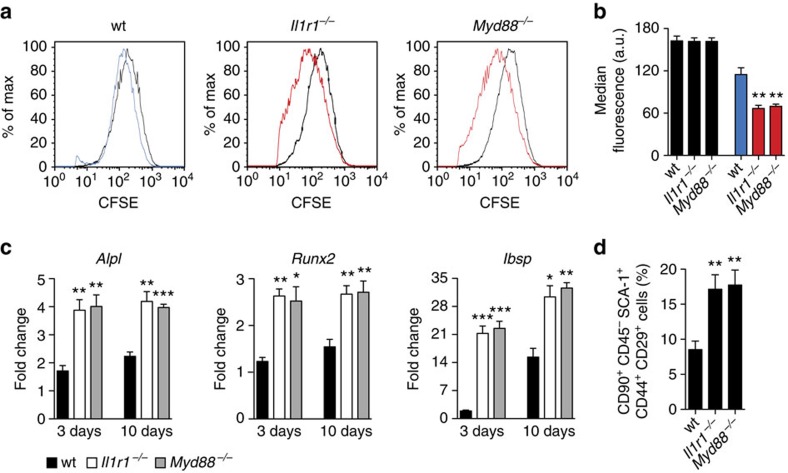
Transplanted MSC functions and mobilization of MSC-like cells are inhibited by IL-1R1/MyD88 signalling. (**a**–**c**) Carboxyfluorescein succinimidyl ester (CFSE)-labelled MSCs from wt, *Il1r1*^−/−^ or *Myd88*^−/−^ mice were delivered into wt mouse calvarial defects (5 mm diameter) within a fibrin matrix. After 7 days, partially remodelled fibrin matrices were removed from the defect and digested to recover cells. (**a**) Difference in proliferation between wt, *Il1r1*^−/−^ and *Myd88*^−/−^ MSCs was determined by comparing their fluorescence intensity using flow cytometry. MSC proliferation is inversely proportional to the fluorescence intensity. Representative histogram plots are shown. Black curves represent cells cultured *in vitro* with 1% serum (no proliferation control). (**b**) Median fluorescence of MSCs 7 days after implantation. Data are means±s.e.m. (*n*=3). **P<0.01; Student's *t*-test. (**c**) CFSE-labelled MSCs were sorted by fluorescence-activated cell sorting and further cultured in osteogenesis induction medium. Expression of osteoblast-specific genes was determined by quantitative PCR, after 3 and 10 days. Fold changes in gene expression relative to MSCs cultured in normal medium are shown. *Alpl*, alkaline phosphatase; *Runx2*, runt-related transcription factor 2; *Ibsp,* integrin-binding sialoprotein. (**d**) Calvarial defects in wt, *Il1r1*^−/−^ or *Myd88*^−/−^ mice were treated with fibrin matrices containing PDGF-BB (1 μg). After 7 days, fibrin matrices were recovered and digested to recover cells. Percentages of MSC-like cells (CD90^+^, CD45^−^, Sca-1^+^, CD44^+^ and CD29^+^ cells) recruited within the matrices were analyzed by flow cytometry. Data are means±s.e.m. (*n*=6 per condition). *P<0.05, **P<0.01, ***P<0.001; Student's *t*-test.

Then, we examined whether proliferation and osteoblastic differentiation of MSCs are also inhibited by IL-1R1/MyD88 signalling *in vivo*. As a model, carboxyfluorescein succinimidyl ester (CSFE)-labelled MSCs from *Il1r1*^−/−^, *Myd88*^−/−^, or wt mice were delivered into calvarial defects with a fibrin matrix and the labelled cells were recovered 7 days later using fluorescence-activated cell sorting. *Myd88*^−/−^ and *Il1r1*^−/−^ MSCs showed significantly less florescence (Fig. 5a,b; Supplementary Fig. 13), indicating that they proliferated more than wt MSCs. In addition, expression of osteoblast differentiation markers (alkaline phosphates, runt-related transcription factor 2 and integrin-binding sialoprotein) was significantly higher in *Myd88*^−*/*−^ and *Il1r1*^−*/*−^ MSCs compared with wt MSCs (Fig. 5c), demonstrating that differentiation of transplanted MSCs towards osteoblasts is impaired by IL-1R1/MyD88 signalling. In addition, we examined if mobilization of stem/progenitor cells following tissue injury is also affected by IL-1R1/MyD88 signalling. As a model, calvarial defects in *Il1r1*^−/−^*, Myd88*^−/−^ or wt mice were treated with a fibrin matrix containing PDGF-BB—a very potent recruiting factor for stem/progenitor cells^35^. After 7 days, the cells recruited in the remodelled fibrin matrix were analysed by flow cytometry. The percentage of MSC-like cells expressing typical surface markers of MSCs (CD45^−^, CD90^+^, CD44^+^, Sca-1^+^ and CD29^+^) was twice higher in *Il1r1*^−*/*−^ and *Myd88*^−/−^ mice compared with wt mice (Fig. 5d; Supplementary Fig. 14).

### IL-1β impairs the Akt/GSK-3β/β-catenin pathway in MSCs

**Figure 6:**
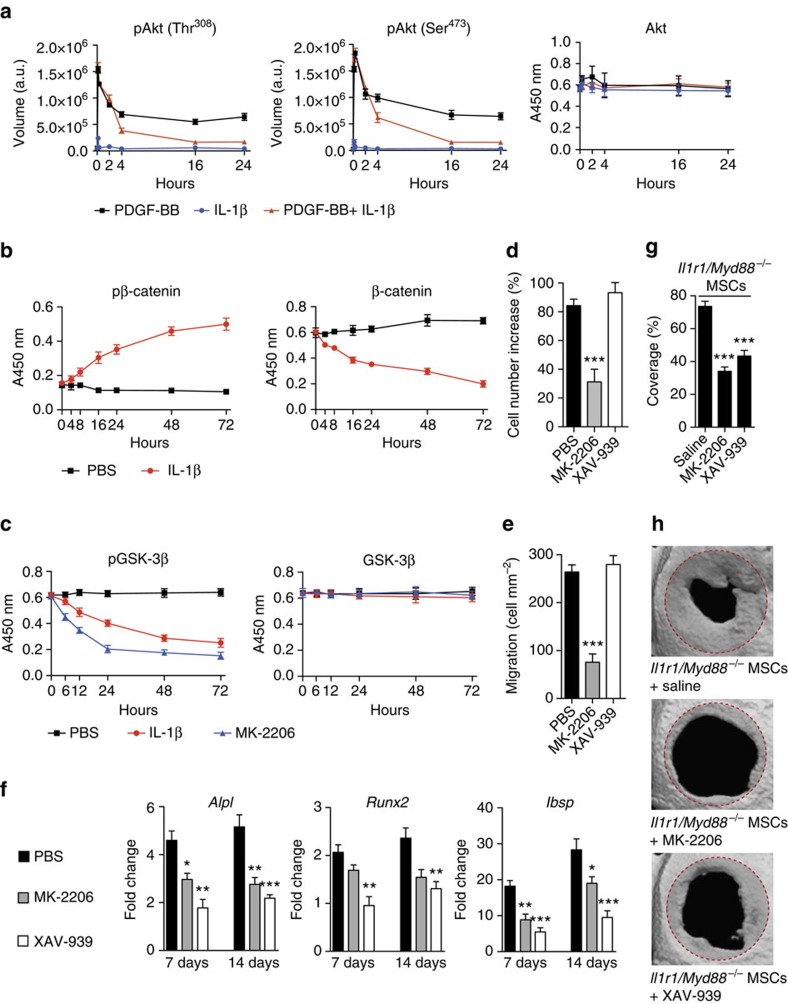
IL-1R1/MyD88 signalling impairs MSC functions via inhibition of the Akt/GSK-3β/β-catenin pathway. (**a**) MSCs were stimulated with PDGF-BB (5 ng ml^−1^), IL-1β (1 ng ml^−1^) or both. Phosphorylation of Akt at Thr^308^ and Ser^473^ was quantified using an antibody array. Akt was measured by enzyme-linked immunosorbent assay. (**b**,**c**) MSCs were treated with IL-1β (1 ng ml^−1^). Phosphorylated β-catenin (pβ-catenin), total β-catenin, phosphorylated GSK-3β (pGSK-3β) and total GSK-3β were quantified using enzyme-linked immunosorbent assay. (**d**) MSC proliferation was stimulated by PDGF-BB (5 ng ml^−1^) with PBS, Akt inhibitor (MK-2206, 10 μM), or β-catenin inhibitor (XAV-939, 10 μM). After 72 h, cell number increase was measured. (**e**) Migration of MSCs through a migration transwell was induced by PDGF-BB (5 ng ml^−1^) with PBS, MK-2206 (10 μM), or XAV-939 (10 μM). After 6 h, the number of cells per square millimetre that passed through the transwell was counted. (**f**) MSCs in osteogenesis induction medium were treated with PBS, MK-2206 (10 μM), or XAV-939 (10 μM). After 7 and 14 days, expression of osteoblast-specific genes was determined by quantitative PCR. Fold changes in gene expression relative to MSCs cultured in normal medium are shown. *Alpl*, alkaline phosphatase; *Runx2*, runt-related transcription factor 2; *Ibsp,* integrin-binding sialoprotein. For **a**–**f** data are means±s.e.m. (*n*≥3). **P<0.01, ***P<0.001; Student's *t*-test. (**g**,**h**) Critical size calvarial defects (5 mm diameter) in mice were treated with *Il1r1*/*Myd88*^−/−^ MSCs co-delivered in fibrin matrix with saline, MK-2206 (1 mM), or XAV-939 (1 mM). Four weeks after treatment, bone regeneration was measured by microCT as coverage of the defect. Data are means±s.e.m. (*n*=6 per condition). ANOVA with Bonferroni *post hoc* test for pair-wise comparisons; ***P<0.001. Representative calvarial reconstructions are shown in **h**. Original defect area is shaded with a red dotted outline. ANOVA, analysis of variance.

To gain insights on the molecular mechanism by which IL-1R1/MyD88 signalling inhibits MSC proliferation and migration, we used an antibody array to analyse the phosphorylation state of key intercellular signalling molecules following co-stimulation with IL-1β and PDGF-BB (Supplementary Fig. 15). Among the proteins tested, the strongest phosphorylation signal induced by PDGF-BB was for the serine/threonine kinase Akt. In contrast, Akt phosphorylation at Thr^308^ and Ser^473^ considerably decreased over time, when cells were co-stimulated with IL-1β (Fig. 6a), indicating that IL-1R1/MyD88 signalling inhibits the Akt pathway. Then, since Wnt/β-catenin signalling is critical for the differentiation of MSCs into osteoblasts^36^,^37^, we investigated if IL-1R1/MyD88 signalling disturbs this pathway, by analysing the phosphorylation state of β-catenin following stimulation with IL-1β. IL-1β induced β-catenin phosphorylation, indicating that β-catenin was undergoing degradation. Confirming β-catenin degradation, the total concentration of β-catenin decreased gradually over time (Fig. 6b). In addition, we examined whether β-catenin degradation induced by IL-1R1/MyD88 signalling is linked to a decrease of Akt activity. We focused on GSK-3β, which is a kinase within the β-catenin destruction complex, because Akt inactivates GSK-3β by phosphorylation of Ser^9^ (ref. 38). In MSCs that were stimulated with IL-1β, the concentration of phosphorylated GSK-3β gradually decreased over time (Fig. 6c), indicating that IL-1R1/MyD88 signalling inhibits the Akt/GSK-3β/β-catenin singling pathway.

To verify that Akt activity and β-catenin directly contribute to MSC proliferation, migration, differentiation and MSC-driven bone regeneration, we used specific inhibitors for Akt and β-catenin, respectively, MK-2206 and XAV-939. MK-2206 directly inhibits Akt phosphorylation at Ser^473^ and Thr^308^, while XAV-939 promotes β-catenin degradation through stabilization of axin^39^. Firstly, both inhibitors were confirmed to be effective in MSCs (Supplementary Fig. 16). Then, we found that Akt inhibition by MK-2206 significantly inhibits proliferation, differentiation and migration of MSCs (Fig. 6d–f). Moreover, similarly to the effect IL-1β, MK-2206 induced the dephosphorylation of GSK-3β (Fig. 6c). Promotion of β-catenin degradation by XAV-939 had no significant effect on MSC proliferation (Fig. 6d) and migration (Fig. 6e), but significantly reduced MSC differentiation into osteoblasts (Fig. 6f). Lastly, we verified that Akt and β-catenin pathways are important for bone regeneration stimulated by MSC delivery. As a model, we treated calvarial defects with *Il1r1*/*Myd88*^−/−^ MSCs co-delivered with MK-2206 or XAV-939 and we analysed regeneration after 4 weeks. Since *Il1r1*/*Myd88*^−/−^ MSCs are not responsive to IL-1, their Akt activity and β-catenin level were affected by the inhibitors, but not by IL-1 present in the defect microenvironment. The regeneration induced by *Il1r1*/*Myd88*^−/−^ MSCs co-delivered with inhibitors was significantly lower compared with regeneration-induced *Il1r1*/*Myd88*^−/−^ MSCs delivered alone (Fig. 6g,h), showing that both Akt and β-catenin pathways are critical for MSC-driven bone regeneration.

### Inhibition of IL-1R1/MyD88 signalling promotes regeneration

**Figure 7:**
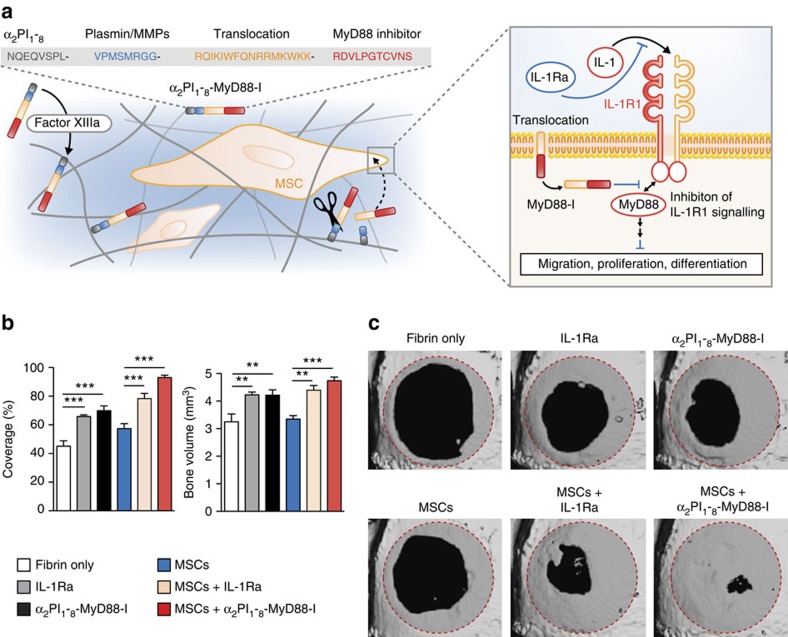
Inhibition of IL-1R1/MyD88 signalling promotes MSC-driven bone regeneration. (**a**) Design of the MyD88 inhibitor peptide (α_2_PI_1-8_-MyD88-I). A fibrin-binding sequence derived from α_2_-PI_1-8_ (NQEQVSPL, in grey) is followed by a plasmin/matrix metalloproteinase-sensitive sequence (VPMSMRGG, in blue), a membrane translocation sequence (RQIKIWFQNRRMKWKK, in orange) and the MyD88 inhibitor peptide (RDVLPGTCVNS, in red). The peptide is covalently crosslinked into fibrin fibres (in grey) during the natural polymerization process of the matrix via the transglutaminase activity of factor XIIIa. Then, following matrix remodeling, the peptide is released by proteases (represented as scissors), translocates into cells, and ultimately inhibits IL-1R1/MyD88 signalling. (**b**,**c**) Critical size calvarial defects (5 mm diameter) in mice were treated with or without MSCs delivered by a fibrin matrix. Fibrin matrices were functionalized with IL-1Ra (1 μg) or α_2_PI_1-8_-MyD88-I (4 μg). Eight weeks after treatment, bone regeneration was measured by microCT as coverage of the defect and bone volume. Data are means±s.e.m. (*n*=6 per condition). ANOVA with Bonferroni *post hoc* test for pair-wise comparisons; **P<0.01, ***P<0.001. Representative calvarial reconstructions are shown in **c**. Original defect area is shaded with a red dotted outline. ANOVA, analysis of variance.

Since we found that IL-1R1/MyD88 signalling represses the regenerative capacities of MSCs, we thought to inhibit this pathway to create a pro-regenerative niche supporting endogenous and transplanted stem cells. As a proof of concept, we engineered a cell delivery system based on fibrin matrix functionalization with IL-1R1/MyD88 signalling inhibitors. We chose two different strategies: (i) delivering the natural inhibitor of IL-1R1, IL-1Ra; (ii) delivering a MyD88 inhibitory peptide, RDVLPGTCVNS (MyD88-I)^40^, which inhibits MyD88 homodimerization. To deliver MyD88-I accurately, MyD88-I was engineered to be covalently crosslinked into fibrin matrix and to translocate into cells. A fibrin-binding sequence derived from α_2_-plasmin inhibitor^41^ (α_2_-PI_1-8_, NQEQVSPL) was added at the N terminus of the peptide, followed by a plasmin/matrix metalloproteinase-sensitive sequence^42^ (VPMSMRGG) and a membrane translocation sequence derived from antennapedia/homeobox protein^43^ (RQIKIWFQNRRMKWKK). Therefore, the peptide is covalently crosslinked into fibrin, during the natural polymerization process of the matrix^41^. Then, following matrix remodelling, the peptide is released by proteases, translocates into cells and ultimately inhibits IL-1R1 signalling (Fig. 7a). First, we confirmed that the engineered peptide could inhibit IL-1R1/MyD88 signalling, as shown by its ability to significantly impair the release of cytokines from MSCs following stimulation with IL-1β (Supplementary Fig. 17). Next, we used the critical calvarial defect model again for testing the cell delivery system, since this versatile model allows for evaluation of biomaterials and bone tissue engineering approaches within a reproducible orthotopic site^25^,^44^. Bone defect were treated with or without MSCs co-delivered with IL-1Ra (1 μg) or α_2_PI_1-8_-MyD88-I (4 μg). Analysis of bone regeneration after 2 month revealed that delivering IL-1Ra or α_2_PI_1-8_-MyD88-I alone improves bone regeneration compared with treatment with fibrin only (Fig. 7b,c). Strikingly, treatment with MSCs co-delivered with IL-1Ra or α_2_PI_1-8_-MyD88-I led to a marked increase of bone tissue deposition as to treatment with MSCs only, with no signs of bone overgrowth and yielding coverage at 78% and 93%, respectively (Fig. 7b,c).

## Discussion

Tissue injury and the following healing process are almost always accompanied with an immune response. Therefore, understanding the immune regulations of tissue repair and regeneration could be fundamental for designing effective regenerative therapies^1^,^2^. Particularly, the regenerative capacities of endogenous and transplanted stem cells could rely on the inflammatory/immune microenvironment at the injury site^1^,^2^,^23^,^45^,^46^. For example, it has been shown that recipient T cells negatively regulate bone formation by autologous MSCs, in the mouse^45^. Moreover, MSC-mediated bone regeneration in the rat has been enhanced, by inhibiting of NF-κB, which is a major transcription factor regulating both the innate and adaptive immune response. As another example, we recently found that self-RNA released after radiation-induced injury promote crypt stem cells death through TLR3 signalling^23^. Since tissue injury and the healing response usually lead to the release of endogenous danger signals such as TLR and IL-1R1 ligands^10^,^11^, and because TLRs and IL-1R1 have been shown to influence the repair of several tissues^13^,^14^,^15^,^16^,^17^,^18^,^19^,^20^,^21^,^22^,^23^,^47^, our initial motivation was to determine the impact of TLR/IL-1R1 signalling during bone regeneration.

While some TLRs have been shown to have a role in the repair of certain organs including brain^16^,^19^,^20^, heart^13^,^47^, liver^14^, kidney^15^ and skin^22^, all TLR knockout mice that we tested displayed normal bone regeneration in specific pathogen-free conditions. In contrast, MyD88- and IL-1R1-deficient mice exhibited better bone regeneration, indicating that IL-1R1/MyD88 signalling negatively regulates bone regeneration. Since IL-1R1/MyD88 signalling is well known to modulate the activity of immune cells, we first suspected that this signalling was inhibiting bone regeneration by acting on the immune cells mobilized after injury. However, using the chimeric mouse model where bone marrow-derived immune cells and osteoclasts are deficient for MyD88, we found that IL-1R1/MyD88 signalling inhibits bone regeneration by acting on tissue-resident cells except osteoclasts. An ideal way to determine if IL-1R1/MyD88 signalling negatively regulated the regenerative capacity of MSCs *in vivo* would have been to analyse bone regeneration in a mouse having only these cells deficient for IL-1R1 or MyD88. Unfortunately, due to the lack of very specific markers for MSCs, there are currently no accurate methods to create such conditional knockout mouse. Therefore, as a model to explore whether IL-1R1/MyD88 signalling inhibits MSC-driven bone regeneration, we compared bone healing stimulated by the local delivery of bone-derived MSCs from wt, *Il1r1*^−/−^ or *Myd88*^−/−^. We found that a much better regeneration is induced by *Il1r1*^−/−^ or *Myd88*^−/−^ MSCs, indicating that IL-1R1/MyD88 signalling inhibits bone regeneration induced by MSCs.

Among IL-1R1 ligands^26^ (IL-1α, IL-1β and IL-1Ra), we observed that IL-1β and IL-1Ra are present at relatively high concentration in the defect during the first 10 days following calvarial injury, while the concentration of IL-1Ra drastically increases afterward. This observation suggests that the IL-1R1/MyD88 signalling inhibiting bone regeneration in this model occurs for at least 1 week post injury. Interestingly, the concentration of IL-1β was about twice lower in calvarial defects of mice having a much lower number monocytes/macrophages, showing that these immune cells significantly contribute to the presence of IL-1β. Nevertheless, other cell types such as recruited neutrophils, mesenchymal cells and endothelial cells probably contribute to the presence of IL-1β in the defect microenvironment. In addition, in the case of transplanted MSCs, the stem cells could also release IL-1β. Importantly, we found that IL-1β significantly impairs colony formation of freshly isolated (primary) MSCs, as well as their differentiation towards osteoblasts. Although MSCs phenotype and proprieties can change during *in vitro* expansion, a strong inhibitory effect of IL-1β was also observed in cultured (expended) MSCs, indicating that IL-1β affects both primary and *in vitro*-expanded MSCs. Notably, while primary bone-derived MSCs and primary bone marrow-derived MSCs were reported to differ in stem cells antigen-1 and nestin expression, c.f.u.-F and osteoblastic differentiation potential^48^,^49^, the inhibitory effects of IL-1R1/MyD88 signalling on bone-derived MSC and bone marrow-derived MSC were the same. IL-1β also inhibited proliferation, migration and differentiation of osteoblasts, but the inhibitory effects of IL-1β on osteoblasts were less strong, compared with MSCs, suggesting that IL-1R1/MyD88 signalling most likely inhibits bone regeneration by acting principally on stem/progenitor cells. Tracing the fate of transplanted wt, *Myd88*^−/−^ and *Il1r1*^−/−^ MSCs, we could demonstrate that both proliferation and osteoblastic differentiation of MSCs delivered in bone defect are impaired by IL-1R1/MyD88 signalling. Moreover, when we examined the mobilization of stem/progenitor cells following bone injury, *Myd88*^−/−^ and *Il1r1*^−/−^ mice—having no cells responsive to IL-1β—mobilized a higher percentage of cells having typical MSC markers. The cell population detected within the defects most likely contained MSCs, while other cell types that also express MSC markers such as pericytes^50^ could have been detected. Still, IL-1R1/MyD88 signalling probably inhibits the mobilization of MSCs in the defect, since IL-1β strongly inhibits MSC migration *in vitro*, and because *Il1r1*^−/−^ and *Myd88*^−/−^ mice recruit more MSC-like cells after bone injury.

Exploring the signalling mechanism by which IL-1β inhibits MSC regenerative functions, we first found that IL-1β promotes the dephosphorylation of Akt. Therefore, since Akt is a central node in cell signalling downstream many growth factor receptors such as PDGF and fibroblast growth factor receptors^38^, IL-1R1/MyD88 signalling probably attenuates MSC response to most Akt-dependent growth factors. Secondly, we found that IL-1β induces β-catenin phosphorylation and degradation, suggesting that IL-1R1/MyD88 directly impairs canonical Wnt signalling, which is critical for MSC differentiation into osteoblasts^36^,^37^. Interestingly, these findings are in line with another study demonstrating that NF-κB—which is activated by IL-1R1 signalling^26^—promotes β-catenin degradation in MSCs and impairs their differentiation into osteoblasts^51^. Moreover, we found that β-catenin degradation induced by IL-1β is linked to an increase of active GSK-3β (unphosphorylated) via a decrease of active Akt (unphosphorylated). Taken together, our data show that IL-1R1/MyD88 signalling inhibits the Akt/GSK-3β/β-catenin singling pathway in MSCs. Yet, the detailed molecular mechanism by which this signalling pathway is inhibited still needs to be determined. For instance, IL-1β could stimulate the expression of phosphatases specific for Akt or induce the expression of proteins involved in β-catenin degradation. Interestingly, it has been shown that NF-κB activation in MSCs promotes β-catenin ubiquitination and degradation through induction of E3 ubiquitin-protein ligase Smurf1 and Smurf2 (ref. 51), giving some insights about possible mechanisms. We could also show that the Akt and β-catenin pathways directly contribute to MSC proliferation, migration and differentiation, using potent and selective inhibitors for these molecules. As expected, Akt activity was critical for MSC migration, proliferation and differentiation, since these processes are known to be dependent on Akt^38^,^52^. On the other hand, β-catenin is probably weakly involved in MSC proliferation and migration, since β-catenin inhibition principally affected MSC differentiation into osteoblasts. Nevertheless, Akt and β-catenin pathways have been shown to positively regulate bone healing^52^,^53^. By co-delivering the inhibitors *in vivo*, we were also able to observe that both pathways are essential for bone regeneration induced by MSC delivery.

After having revealed that IL-1R1/MyD88 signalling inhibits the regenerative potentials of MSCs, we engineered a proof of concept MSC delivery system based on fibrin matrix functionalization with IL-1R1/MyD88 signalling inhibitors. As a first inhibitor, we chose IL-1Ra, since the protein is the natural antagonist of IL-1R1. Moreover, just as fibrin, IL-Ra is clinically relevant, since the molecule is clinically available for the treatment of rheumatoid arthritis. As a second inhibitor, we chose to deliver α_2_PI_1-8_-MyD88-I, because synthetic peptide delivery could be more cost effective than recombinant protein delivery. Strikingly, we found that delivering MSCs with IL-1Ra or α_2_PI_1-8_-MyD88-I led to a large increase of bone tissue deposition as to treatment with MSCs only. Interestingly, α_2_-PI_1-8_-MyD88-I was slightly more efficient than IL-1Ra, yielding coverage near to 100%. This difference could be explained by the higher concentration of the peptide used compared with IL-1Ra (20 versus 2 μM) and because α_2_-PI_1-8_-MyD88-I was engineered to be released in a controllable manner following fibrin matrix remodelling. Yet, the delivery of a fibrin-binding version of IL-1Ra or the co-delivery of IL-1Ra with α_2_-PI_1-8_-MyD88-I at lower doses could be very effective. In addition, while the calvarial critical size defect is a well-accepted model that is usually considered as translationally relevant^25^,^44^, further tests will be required to predict the clinical potential of IL-1R1/MyD88 signalling inhibition. Experiments in more realistic models would also confirm the physiological relevance of the findings. Typically, these tests would be done using critical size bone defect models in larger animal closer to human such as dogs, goats or sheep, since success in rodents is not enough to open the door for human trials^24^,^54^.

Most importantly, we demonstrated that MSCs delivered without an inhibitor of IL-1R1/MyD88 signalling could not promote regeneration, reflecting that one cannot assume that stem/progenitor cells automatically stimulate tissue healing. In other words, applying stem cells to damaged tissue with little or no attempt to prepare and engineer the regenerative niche is unlikely to promote engraftment^2^. Indeed, several studies have shown that MSCs from different sources are inefficient in promoting bone regeneration^45^,^51^,^55^,^56^. Moreover, regenerative medicine therapies based on stem cells did not yet demonstrate real effectiveness in the clinic^4^,^57^,^58^. Our poor understanding of how transplanted cells are controlled by the host tissue is probably one the main reasons underlying this modest translation to the clinic. On the front line, understanding and controlling the immune microenvironment at the delivery site could be the key to improve the efficacy of stem cell-based therapies^2^. Here, we revealed that the innate immune response via IL-1R1/MyD88 signalling impairs bone regeneration and the regenerative capacity of MSCs. By using a simple stem cell delivery integrating an inhibitor of IL-1R1/MyD88 signalling, we could considerably improve MSC-driven bone regeneration. Furthermore, such a strategy may be adapted into cell delivery systems using other biomaterial matrices or for MSC-based regenerative strategies targeting other tissues. This work highlights the crucial role of the innate immune response in modulating the regenerative capacity of stem cells and the importance of integrating a control of the host immune microenvironment into regenerative strategies.

## Methods

### Animals

All animals were kept under specific pathogen-free conditions. Wt *C57BL/6* mice were purchased from SLC Japan. *Myd88*^−/−^ (ref. 59), *Trif*^−/−^ (ref. 60), *Tlr1*^−/−^ (ref. 61), *Tlr5*^−/−^ (ref. 62), *Tlr7*^−/−^ (ref. 63), *Tlr2/4/9*^−/−^ (refs 64, 65, 66) and *Il1r1*^−/−^ (ref. 59) mice were backcrossed onto a *C57BL/6* background for more than eight generations. All animal experiments were performed with the approval of the Animal Research Committee of the Research Institute for Microbial Diseases (Osaka University).

### Calvarial defect model

Mice used for surgery were 10–12 weeks old. Mice were anaesthetized with isoflurane. The top of their head was shaved and a longitudinal incision was performed to reveal the skull. Bone tissue was exposed by retracting the soft tissues. Using a drill, two craniotomy defects (5 mm diameter) were created in the parietal bones of the skull on each side of the sagittal suture line. The defects were washed with saline and covered with a fibrin matrix polymerized atop the dura (40 μl per defect, 10 mg ml^−1^ fibrinogen (Enzyme Research Laboratories), 2 U ml^−1^ of thrombin (Sigma-Aldrich), 5 mM CaCl_2_, 25 μg ml^−1^ aprotinin (Roche), 2 × 10^6^ MSCs). For Akt and β-catenin inhibition experiments, MK-2206 or XAV-939 were added to the MSC mix to have a final concentration of 1 mM per fibrin matrix. Then, the soft tissue was closed with stitches. As a painkiller, mice received a subcutaneous injection of Tramdol (100 mg kg^−1^).

### Microcomputed tomography

Skulls were scanned with a Scan-Xmate RB080SS110 system (Comscan Techno Co., Ltd) for screening and with a microCT 40 (Scanco Medical AG) operated at energy of 70 kVp and intensity of 145 ms for detailed measurements. Scans were performed at high-resolution mode resulting in a nominal isotropic resolution of 30 μm. After reconstruction, a 3D Gaussian filter (sigma 1.2, support 1) was applied to all images. Bone was segmented from background using a global threshold of 22.4% of maximum grey value. Afterwards, cylindrical masks were placed manually at the defects. Bone volume within these masks was calculated using a standardized procedure developed for quantitative bone morphometry^67^. Coverage was calculated on a dorso-ventral projection of the cylindrical area^68^.

### Chimeric mice

Bone marrow cells (1 × 10^7^) from 5-week-old *MyD88*^−/−^ mice or wt mice were intravenously injected into lethally irradiated 4-weeks-old recipient wt mice. Mice were placed on 10 ml l^−1^ neomycin sulfate for 2 weeks post radiation. After 6 weeks, most of bone marrow-derived immune cells and osteoclasts in bone tissue should lack MyD88 (ref. 69). Mice were used for cranial surgery 8 weeks after bone marrow transplantation as described for the calvarial defect model. After 4 weeks, skulls were analysed by microCT and bone marrow from femurs was harvested to confirm the absence of MyD88 in chimeric mice. Bone marrow-derived cells were lysed with tissue protein extraction reagent (T-PER, Thermo Scientific) supplemented with protease inhibitor cocktail (one tablet of protease inhibitor cocktail (Roche) for 10 ml). The lysed cells were centrifuge at 5,000*g* for 5 min and the supernatant was analysed using ELISA for MyD88 (MyBioSource).

### Release of cytokines and inflammatory-associated molecules following bone injury

Calvarial defect (4 mm diameter) were treated with a fibrin matrix as described for the calvarial defect model. After 1, 3, 6, 10 and 15 days, the partially remodelled matrix and the bone tissue surrounding the defect (1 mm farther) was collected. As a control a 5 mm diameter calvarial bone tissue was collected (day 0). Fibrinous matrices and tissue samples were incubated in 1 ml of tissue protein extraction reagent (T-PER, Thermo Scientific) containing protease inhibitors (one tablet of protease inhibitor cocktail (Roche) for 10 ml) and homogenized with a tissue homogenizer. Tissue lysates were incubated 1 h at 37 °C and centrifuged at 5,000*g* for 5 min. Supernatants were stored at −80 °C. Cytokines were detected using an antibody array (Mouse Cytokine Array Panel A, R&D Systems) and by ELISA (Mouse IL-1 beta/IL-1F2 DuoSet and Mouse IL-1ra/IL-1F3 DuoSet, R&D Systems) according to the manufacturer's instructions. For the antibody array, 400 μl of lysate was used. The chemiluminescent signals were detected using ImageQuant LAS 4000 and quantified with ImageQuant TL software (GE Healthcare Life Sciences). A measured volume over 1 × 10^6^ was considered as a positive signal.

### Monocytes/macrophages depletion

One day before surgery, 200 μl of clodronate liposomes or empty liposomes (Anionic liposomes, FormuMAx Scientific Inc.) was intravenously injected in wt mice. Additional 50 μl of clodronate liposomes or empty liposomes was intravenously injected right before surgery and every 2 days until day 6. The concentration of IL-1β into the defect was measured by enzyme-linked immunosorbent assay (ELISA) as described above. The efficiency of monocytes/macrophages depletion was checked by measuring the percentage of CD11b^+^ cells in the spleen by flow cytometry (anti-mouse CD11b, Biolegends).

### Isolation of bone-derived MSCs

Long bones of arms and legs of wt, *Myd88*^−/−^ and *Il1r1*^−/−^ mice (6–8 weeks old) were detached from the body trunk. All muscles and cartilages were removed. The bones were rinsed with PBS and cut into three pieces, to have one diaphysis part and two metaphyses/epiphyses parts. The bone marrow within the medullary cavity of the diaphyses was flushed out using a 27-Gauge needle attached to a 10 ml syringe filled with α-MEM containing 1% fetal bovine serum (FBS). Bones pieces, including diaphyses and metaphyses/epiphyses were washed with PBS and cut into 1–3 mm^3^ pieces. Bone chips were transferred in a 25 cm^2^ cell culture-treated flask with 3 ml of α-MEM medium containing 2 mM L-glutamine, 100 mg ml^−1^ penicillin/streptomycin, 10% FBS, and 1 mg ml^−1^ collagenase type II (Life Technologies). Bone chips were digested for 1 h at 37 °C to release most of hematopoietic cells on the inner interface of the bones. Then, the bone chips were washed with MSC culture medium (α-MEM, 2 mM L-glutamine, 100 mg ml^−1^ penicillin/streptomycin, 10% heat inactivated MSC-certified FBS (Gibco)) and incubated at 37 °C with 5% CO_2_ in 6 ml of MSC culture medium. Bone chips were kept in culture for 6 days with one medium change at day 3. Then, the MSCs that have migrated out of the bone chips and formed colonies were detached using trypsin/EDTA. Cells were splitted at a ratio of 1:3 (passage 0) and the bone chips were reseeded together for allowing MSCs to continue migrating out for 3 more days. Five independent isolates were pulled together and MSCs were further expended for three passages before storage. The expression of MSC-specific surface marker was verified using flow cytometry. MSCs were stained for 15 min in 100 μl of flow cytometry buffer (PBS containing 2% of heat inactivated serum) containing anti-mouse antibodies (Biolegends): CD11, CD14, CD19, CD34, CD45.2, Ly-6 A/E (Sca-1), CD29, CD44, CD75 and CD90.1. Cells were washed twice with flow cytometry buffer and further analysed by flow cytometry (BD FACSCanto II). For cells used in colonies formation assay, bone pieces were gently disrupted using a mortar and pestle, in washing buffer (PBS with 2% FBS and 1 mM EDTA). Disrupted bone was rinsed with PBS containing 2% FBS and 1 mM EDTA until bone fragments turned white. Then, bone fragments were transferred to a 100 mm dish containing a collagenase solution (2 ml of 0.25% collagenase Type I (Stemcell Technologies) in PBS containing 20% FBS). Using a scalpel, bone fragments were cut into small pieces (1–2 mm) and transferred into a 50 ml polypropylene tube with 10 ml collagenase solution. Bone fragments were digested at 37 °C under agitation for 45 min. Then, 30 ml of washing buffered was added and supernatant was filtered through a 70 μm cell strainer. Cells were centrifuged at 300*g* for 10 min and resuspend in 500 μl of medium. Lastly, CD45^+^ cells were depleted (MagCellect Mouse Mesenchymal Stem Cell Isolation Kit, R&D Systems) before colonies formation assays.

### MSC isolation from bone marrow

Long bones of arms and legs of wt (6–8 weeks old) were detached from the body trunk. All muscles and cartilages were removed and the bones were rinsed with PBS. The bone marrow was flushed out using a 27-Gauge needle attached to a 10 ml syringe filled with α-MEM containing 1% FBS. Bone marrow cells were passed through a 70 μm filter mesh and transferred in tissue culture-treated plate at a density of 2.5 × 10^7^ cells per ml^−1^. Cells were incubated at 37 °C with 5% CO_2_ in a humidified chamber for 4 h. Non-adherent cells were removed by changing the medium to culture medium (α-MEM containing 2 mM of L-glutamine, 100 mg ml^−1^ penicillin/streptomycin, and 10% FBS). Thereafter, medium was replaced every 8 h. After 72 h, cells were washed with PBS and new medium was added. Then, medium changed every 3–4 days. After 2–3 weeks, MSC colonies formed were detached using trypsin/EDTA (passage 0). MSCs were expanded until 3 passages. For cells used in colonies formation assay, CD45^+^ cells were depleted (MagCellect Mouse Mesenchymal Stem Cell Isolation Kit, R&D Systems).

### Colony formation assay

Primary cells were seeded in six-well plates (50 bone-derived cells) or 12-well plates (200 bone marrow-derived cells) and cultured in medium (α-MEM, 100 mg ml^−1^ penicillin/streptomycin, 20% FBS) with or without 1 ng ml^−1^ of IL-1β for 12 days. Medium was changed once after 6 days. Then, cells were washed with PBS and stained with an ice-cold crystal violet solution (0.5% in methanol). The number of colony (>50 cells) was counted and their size was measured using ImageJ software. For osteobalstic differentiation of colonies, primary cells (50 bone-derived cells) were seeded in 24-well plate with osteogenesis induction medium (α-MEM with 2 mM of L-glutamine, 10% FBS, 100 mg ml^−1^ penicillin/streptomycin, 50 μM ascorbate-phosphate, 10 mM β-glycerolphophate and 100 ng ml^−1^ of human BMP-2 (R&D Systems)) with or without 1 ng ml^−1^ of IL-1β. After 28 days, cells were fixed with ice-cold methanol and calcified nodules were stained using a calcified nodule staining kit (AK-21; Primary Cell).

### Osteoblast isolation

Calvariae from 3-day-old mice were isolated and further digested in α-MEM containing enzymes (0.1% collagenase type II (Life Technologies), 0.2% dispase (Sigma-Aldrich)) at 37 °C for 20 min in a shaking water bath to release calvarial cells. The supernatant containing released cells was transferred in a new tube, centrifuged at 300 × g, and the pellet was resuspended in medium (α-MEM, 100 mg ml^−1^ penicillin/streptomycin, 10% FBS). The calvariae were digested three more times for a total of four digestions. Digestions 3 and 4 containing osteoblasts were pulled together and transferred in a tissue culture-treated plate at a density of 3 × 10^5^ cells ml^−1^. Calvarial cells were then maintained in culture for 2 weeks in osteogenesis inducing medium (α-MEM with 2 mM of L-glutamine, 10% FBS, 100 mg ml^−1^ penicillin/streptomycin, 50 μM ascorbate-phosphate, 10 mM β-glycerolphophate, 100 ng ml^−1^ of human BMP-2 (R&D Systems)) and stored before use.

### Cell proliferation assays

Proliferation assays were performed as described previously^70^. Briefly, cells were starved 24 h with α-MEM containing 1% FBS. Then, cells were seeded in 96-well plate with α-MEM containing murine PDGF-BB (5 ng ml^−1^, Peprotech) and 1% FBS, or α-MEM containing 10% FBS. Directly after, murine IL-1β (Peprotech) was added at concentrations ranging from 1 to 1,000 ng ml^−1^. For Akt and β-catenin inhibition experiments, MK-2206 (AdooQ Bioscience) or XAV-939 (AdooQ Bioscience) were added instead of IL-1β at a concentration of 10 μM. After 72 h, cell number was quantified using PrestoBlue dye (Invitrogen). Percentage proliferation increases were calculated over basal proliferation (medium with 1% FBS and no PDGF-BB).

### Migration assays

Migration assays were performed as described previously^70^. Briefly, solutions containing murine PDGF-BB (5 ng ml^−1^) with or without 1 ng ml^−1^ of murine IL-1β were added to the bottom side of a collagen type I (C4243, Sigma-Aldrich) coated transwell (8 μm pore size, Millipore). For Akt and β-catenin inhibition experiments, MK-2206 or XAV-939 were added instead of IL-1β at a concentration of 10 μM. Directly thereafter, cells (30,000 cells per transwell) were added to the transwell upper chambers. After 6 h, the numbers of cells that migrated to the bottom side of the membrane were counted. Membranes of each insert were removed and mounted on microscopy glass slides using Vectashield (Vector Laboratories) mounting medium containing DAPI. The number of cells that migrated to the bottom side of the membrane was counted.

### Osteoblastic differentiation assays

Cells were seeded on 24-well plate at 70–80% confluency in osteogenesis inducing medium with or without murine IL-1β (1 ng ml^−1^). For Akt and β-catenin inhibition experiments, MK-2206 or XAV-939 were added instead of IL-1β at a concentration of 10 μM. After 4 days, osteogenesis inducing medium was replaced (without IL-1β). Medium was changed every 3–4 days. Seven or 14 days following stimulation with IL-1β, total RNAs were isolated, using RNeasy Plus Mini Kit (Qiagen) and reverse transcription was performed using ReverTra Ace (Toyobo Co., Ltd.). Quantitative PCR was performed with an ABI PRISM 7500 using TaqMan Assay with the following primers: Alpl Mouse, Mm00475834_m1; Runx2 Mouse, Mm00501580_m1; Ibsp Mouse, Mm00492555_m1; Eukaryotic 18S rRNA Endogenous Control (VIC/MGB Probe, primer limited) (Applied Biosystems). For extracellular matrix mineralization analysis, MSCs were cultured for 28 days in osteogenesis inducing medium. Then, cells were fixed with methanol and calcified nodules were stained using a calcified nodule staining kit.

### Chondrogenic differentiation of MSCs

MSCs were transferred in a 15 ml conical tubes (250,000 cells per tube), centrifuged at 200*g* for 5 min and resuspended in D-MEM/F12. MSCs were centrifuged again and resupended in 0.5 ml of D-MEM/F12 containing 100 mg ml^−1^ penicillin/streptomycin, 1% ITS supplement, 1 × chondrogenic supplement (R&D Systems), with or without murine IL-1β (1 ng ml^−1^). Cells were centrifuged on more time at 200*g* for 5 min to form a pellet. The cap of the tubes were loosen to allow gas exchange and the tubes were incubated upright at 37 °C with 5% CO_2_. The medium was replaced every 3 days (without IL-1β). After 28 days, the spheroids were washed with PBS and fixed with 10% formalin for 60 min. Spheroids were washed twice with water and stained with Alican Blue 8 GX (Sigma-Aldrich, 0.1 mg ml^−1^ in ethanol/acetic acid solution (3:2)) overnight at room temperature in the dark. Spheroids were destained three times with an ethanol/acetic acid solution (3:2) and resuspended in PBS, before imaging.

### Trophic factors secreted by MSCs

MSCs were seeded in six-well plates and cultured until 70–80% confluency. Cells were starved for 24 h with medium containing 1% FBS and further stimulated with PBS or IL-1β (1 ng ml^−1^). After 24 h supernatants were collected and stored at −80 °C. Trophic factors were detected using an antibody array focusing on angiogenic factors (Mouse Angiogenesis Antibody Array, R&D Systems) according to the manufacturer's instructions, using 400 μl of supernatant. The chemiluminescent signals were detected using ImageQuant LAS 4000 and quantified with ImageQuant TL software (GE Healthcare Life Sciences). A measured volume over 1 × 10^3^ was considered as a positive signal.

### Intracellular signalling array

MSCs were seeded in six-well plate and cultured until 60–70% confluency. Cells were starved 24 h with α-MEM containing only 1% FBS. Then, cells were stimulated with murine PDGF-BB (5 ng ml^−1^), murine IL-1β (1 ng ml^−1^), or with both for 10 min to 24 h. Phosphorylation and cleavage of intrecellular signalling molecules were detected using an antibody array (PathScan Intracellular Signalling Array, Cell Signalling) according to the manufacturer's instructions. The chemiluminescent signals were detected using ImageQuant LAS 4000 and quantified with ImageQuant TL software (GE Healthcare Life Sciences).

### Akt signalling assay

MSCs were seeded in six-well plate and cultured until 60–70% confluency. Then, cells were stimulated with murine IL-1β (1 ng ml^−1^) or PBS for 0 to 72 h. For Akt and β-catenin inhibition experiments, MK-2206 or XAV-939 were added instead of IL-1β at concentrations ranging from 0 to 10 μM. Total Akt and phosphorylated Akt were quantified using ELISA (InstantOne ELISA, affimetrix eBioscience) according to the manufacturer's instructions.

### Wnt/β-catenin and GSK-3β signalling assays

MSCs were seeded in six-well plate and cultured until 60–70% confluency. Then, cells were stimulated with murine IL-1β (1 ng ml^−1^) or PBS for 4 to 72 h. For Akt and β-catenin inhibition experiments, MK-2206 or XAV-939 were added instead of IL-1β at a concentration ranging from 0 to 10 μM. Total β-catenin and phosphorylated β-catenin were quantified using ELISA (InstantOne ELISA, affimetrix eBioscience) according to the manufacturer's instructions. Total GSK-3β and phosphorylated GSK-3β were quantified using ELISA (InstantOne ELISA, affimetrix eBioscience) according to the manufacturer's instructions.

### Proliferation of transplanted MSCs

Wt*, Myd88*^−/−^ and *Il1r1*^−/−^ MSCs were cultured until 80–90% confluency. Cells were washed with PBS and incubated with 10 μM of CSFE (Life Technologies) in PBS for 15 min at 37 °C. Thereafter, CSFE solution was removed and replaced with α-MEM containing 10% FBS and further incubated for 30 min at 37 °C. Cells were detached using trypsin/EDTA and transplanted in cranial defect as described for the calvarial defect model (2 × 10^6^ cells per defect within a 40 μl fibrin matrix). As non-proliferating cells control, MSCs were seeded in cell culture plate with α-MEM containing only 1% FBS. As a wt proliferating cells control MSCs were seeded in cell culture plate with α-MEM containing 10% FBS or 10% FBS with 5 ng ml^−1^ of PDGF-BB. Medium was renewed after 4 days. Seven days after transplantation, the partially remodelled fibrinous matrices containing MSCs were removed from the defect and incubated in 1 ml of an enzyme solution (trypsin (10 mg ml^−1^) and collagenase II (1 mg ml^−1^)) for 1 h at 37 °C. Then, the digested matrix was resuspended in 10 ml of medium containing 10% serum, passed through a cell strainer and centrifuged. The cells were resuspended in 1 ml of red blood cell lysis buffer (Sigma-Aldrich), incubated at room temperature for 5 min, and resuspended in 10 ml of flow cytometry buffer. For non-proliferating cells and wt proliferating cells controls, cells were detached from cell culture plate using trypsin/EDTA and resuspended in flow cytometry buffer. Cells were washed twice with flow cytometry buffer and further analysed by flow cytometry (BD FACSCanto II).

### Osteoblastic differentiation of transplanted MSCs

As a non-differentiated control, wt*, Myd88*^−/−^ and *Il1r1*^−/−^ MSCs were seeded in cell culture plate with α-MEM containing 10% FBS for 7 days. At the same time, wt*, Myd88*^−/−^ and *Il1r1*^−/−^ MSCs were labelled with CSFE, transplanted into calvarial defect, and recovered from the defect, as described for the proliferation of transplanted MSCs assay. Cells recovered from the fibrin matrix were sorted using FACS (BD FACSAria II), to isolate CSFE-labelled MSCs. After recovery, MSCs were seeded on 24-well plate at 70–80% confluency in osteogenesis inducing medium. After 3 and 10 days, total RNAs were isolated and quantitative PCR was performed as described for the osteoblastic differentiation assay.

### Mobilization of MSC-like cells

Calvarial defects were treated with fibrin matrix (40 μl) containing 1 μg of murine PDGF-BB, as described for the calvarial defect model. After 7 days, the partially remodelled fibrinous matrices containing endogenous cells were removed from the defect site and incubated in 1 ml of an enzyme solution as described for the proliferation of transplanted MSCs assay. Isolated cells were stained for 15 min in 100 μl of flow cytometry buffer containing antibodies (Biolegends, 1:200 dilution): anti-mouse CD29, anti-mouse Ly-6 A/E (Sca-1), anti-mouse CD90.1, anti-mouse CD45.2, and anti-mouse CD44. Cells were washed twice with flow cytometry buffer and further analysed by flow cytometry (BD FACSCanto II).

### Peptide inhibitor of IL-1R1/MyD88 signalling (α_2_PI_1-8_-MyD88-I)

The peptide α_2_PI_1-8_-MyD88-I (NQEQVSPLVPMSMRGGRQIKIWFQNRRMKWKKRDVLPGTCVNS) was synthesized by GeneScript and verified as ≥95.0% by HPLC. The peptide was further dialyzed against HEPES buffer (20 mM HEPES, 150 mM NaCl, pH 7.5). The capacity of the peptide to inhibit IL-1R1/MyD88 signalling was assessed by monitoring the release of cytokines from MSCs following stimulation with murine IL-1β. MSCs seeded in 24-well at 70–80% confluency were stimulated with 10 ng ml^−1^ of IL-1β together with α_2_PI_1-8_-MyD88-I at increasing concentration (1–100 μg ml^−1^). After 24 h, the concentration of IL-6, CCL2, CXCL1 and CXCL2 released were measured using ELISA (Mouse IL-6, CCL2/JE/MCP-1, CXCL1/KC and CXCL2/MIP-2; DuoSets, R&D Systems).

### MSC delivery system containing inhibitors of IL-1R1/MyD88 signalling

Fibrin matrices with or without wt MSCs were delivered into cranial defects as described for the calvarial defect model (2 × 10^6^ cells per defect). Fibrin matrices (40 μl per defect, 10 mg ml^−1^ fibrinogen, 2 U ml^−1^ of thrombin, 5 mM CaCl_2_, 25 μg ml^−1^ aprotinin) were functionalized with 4 μg of α_2_PI_1-8_-MyD88-I or 1 μg of murine IL-1Ra (R&D Systems). After 8 weeks, bone regeneration was analysed by microCT as described above.

## Additional information

**How to cite this article:** Martino, M. M. *et al*. Inhibition of IL-1R1/MyD88 signalling promotes mesenchymal stem cell-driven tissue regeneration. *Nat. Commun.* 7:11051 doi: 10.1038/ncomms11051 (2016).

## Supplementary Material

Supplementary Information Supplementary Figures 1-17
