# Supplementary material for: Inhibition of IL-1R1/MyD88 signalling promotes mesenchymal stem cell-driven tissue regeneration
Source: Nat Commun. 2016 Mar 22;7:11051. doi: 10.1038/ncomms11051 (PMC4804175; doi:10.1038/ncomms11051)
Supplement: Supplementary Information — Supplementary Figures 1-17 [file ncomms11051-s1.pdf]

Supplementary Information

Inhibition of IL-1R1/MyD88 signaling promotes mesenchymal stem cell–driven tissue regeneration

Mikaël M. Martino[1], Kenta Maruyama[1], Gisela Kuhn[2], Takashi Satoh[1], Osamu Takeuchi[1,3], Ralph Müller[2], Shizuo Akira[1].

[1]WPI Immunology Frontier Research Center, 3-1 Yamada-oka, Suita, Osaka University, Osaka 565-0871, Japan.

[2]Institute for Biomechanics, Leopold-Ruzicka-Weg 4, ETH Zurich, 8093 Zurich, Switzerland.

[3]Institute for Virus Research, 53 Shogoin Kawara-cho, Sakyo-ku, Kyoto University, Kyoto 606-8507, Japan.

Corresponding authors:

Shizuo Akira

sakira@biken.osaka-u.ac.jp

Phone: +81668798302

Mikaël M. Martino

mmartino@ifrec.osaka-u.ac.jp

Phone: +81668798303

Content:

- Supplementary figures 1–17

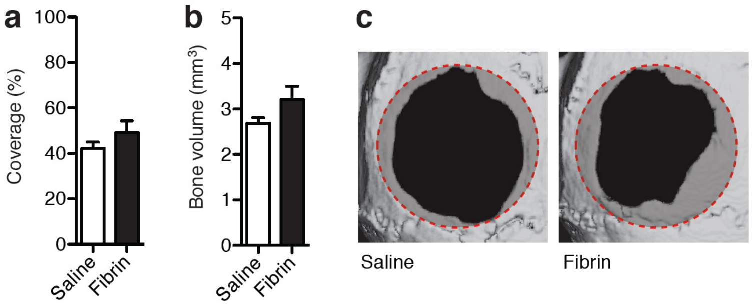

Supplementary figure 1. Effect of fibrin matrix on calvarial bone regeneration. Critical-size calvarial defects (5 mm diameter) in mice were treated with saline or fibrin matrix. (**a, b**) Height weeks after treatment, bone regeneration was measured by microCT as coverage of the defect and bone volume. Data are means ± SEM (n = 6 per condition). No significant differences were found, using Student's t-test. (**c**) Representative calvarial reconstructions. Original defect area is shaded with a red dotted outline. Standard fibrin matrix does not significantly influence calvarial bone regeneration.

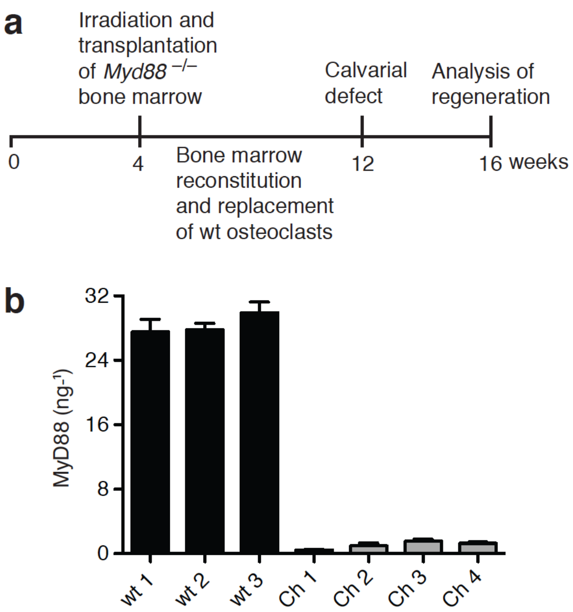

Supplementary figure 2. Chimeric mice lacking MyD88 in bone marrow-derived immune cells and osteoclasts. (**a**) Schedule of bone regeneration analysis in MyD88 chimeric mice. (**b**) Irradiated wt mice received a transplant of bone marrow from wt or *Myd88*$^{-/-}$ mice. The concentrations of MyD88 in bone marrow cells (containing osteoclast precursors) were measured by ELISA, 12 weeks after bone marrow reconstitution. All chimeric mice (Ch 1–4) expressed very low level of MyD88 in bone marrow cells, indicating that most of bone marrow-derived immune cells and osteoclasts lack MyD88. Data are means ± SEM (triplicate per mouse). Of note, irradiated *Myd88*$^{-/-}$ mice having received a transplant of bone marrow from wt mice did not survive bone marrow reconstitution.

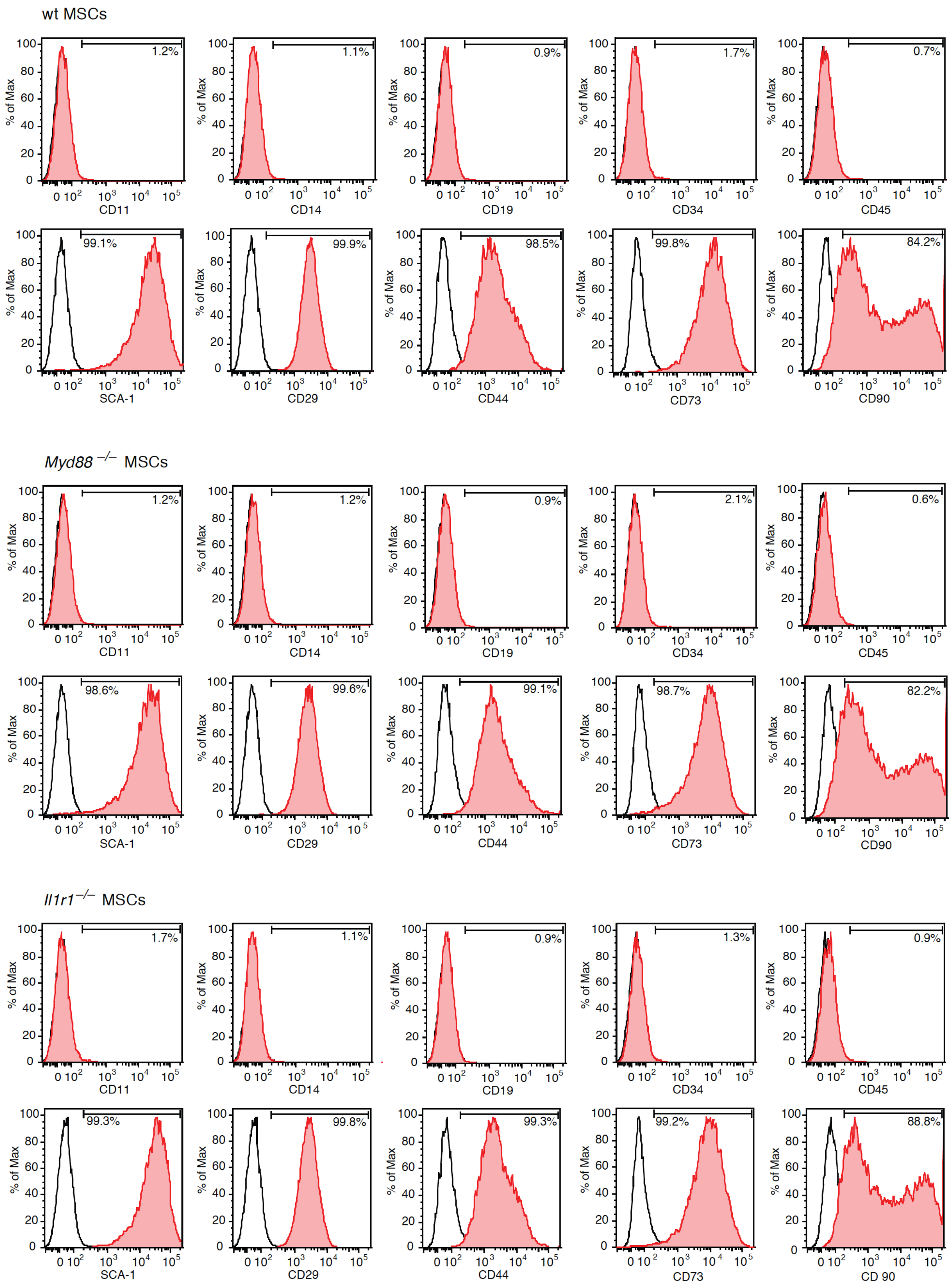

Supplementary figure 3. Surface markers of MSCs. MSCs from wt, *Myd88*$^{-/-}$, and *Il1r1*$^{-/-}$ were isolated from long bones of mice and expanded for 3 passages. Expression of MSC-specific surface markers was verified using flow cytometry. MSC phenotype was confirmed, since cells were CD11^{-}, CD14^{-}, CD19^{-}, CD34^{-}, CD45.2^{-}, Sca-1 (Ly-6A/E)$^{+}$, CD29^{+}, CD44^{+}, CD73^{+}, and CD90.1^{+}.

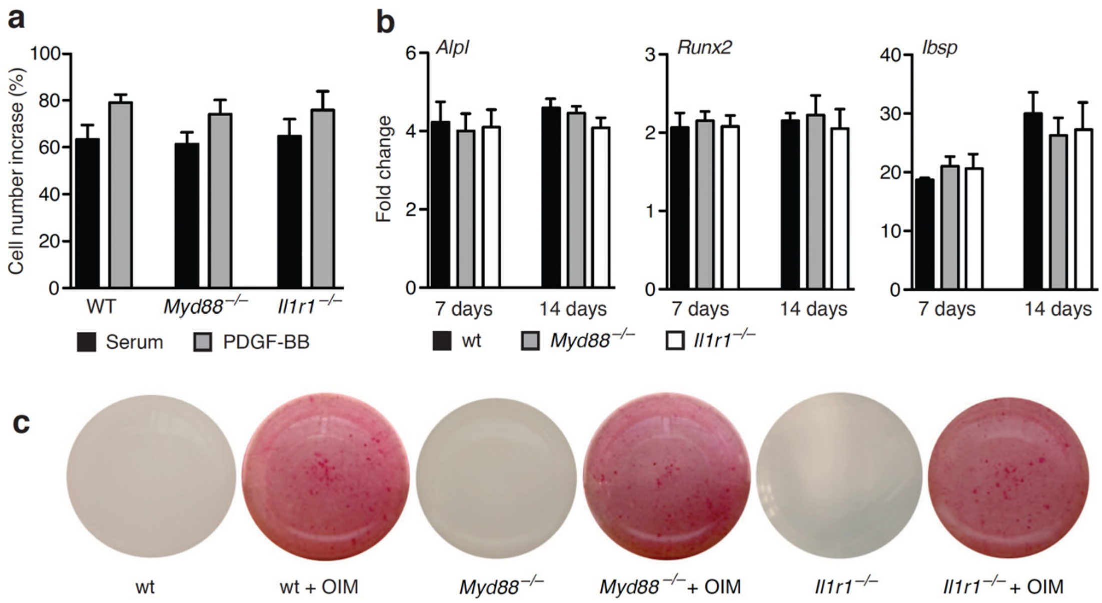

Supplementary figure 4. Proliferation and osteoblastic differentiation of MSCs from wt *Myd88*$^{-/-}$ and *Il1r1*$^{-/-}$ mice. (**a**) MSCs were stimulated with serum or PDGF-BB, and cell number increase was determined after 72 h. (**b**) Osteoblastic differentiation of wt, *Myd88*$^{-/-}$, and *Il1r1*$^{-/-}$ MSCs was stimulated with osteogenesis induction medium containing bone morphogenetic protein-2. Expression of osteoblast-specific genes was determined by quantitative PCR. Fold changes in gene expression relative to MSCs cultured in normal medium are shown. *Alpl*, alkaline phosphatase; *Runx2*, runt-related transcription factor 2; *Ibsp,* integrin-binding sialoprotein. For panels a and b, data are means ± SEM (n = 3). No significant differences were found, using Student's *t*-test. (**c**) Matrix mineralization revealed with alizarin red staining after 28 days of culture with or without osteogenesis induction medium (OIM). Representative wells (2 cm^2) are shown. Wt, *Myd88*$^{-/-}$, and *Il1r1*$^{-/-}$ have equal ability to proliferate and to differentiate into osteoblasts.

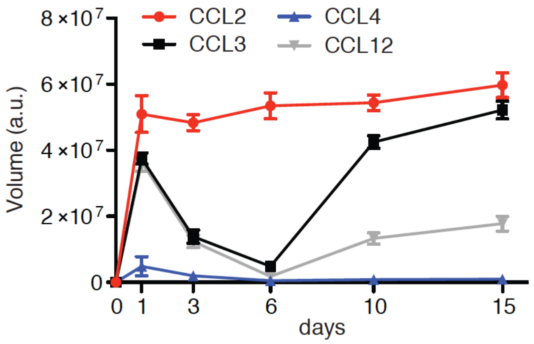

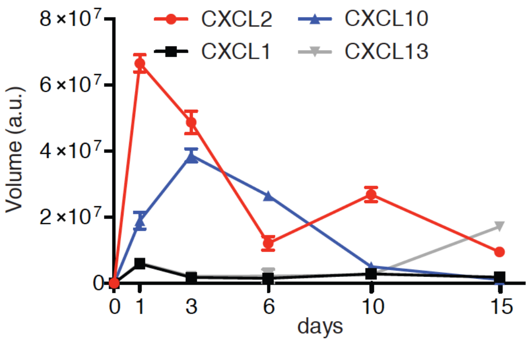

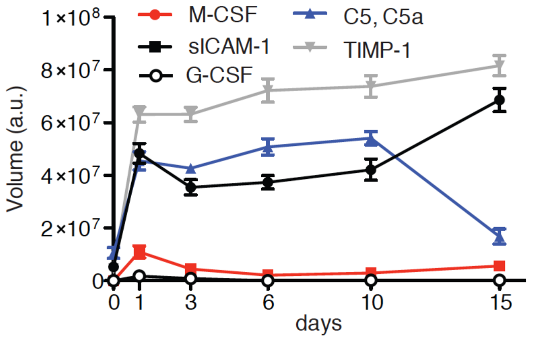

Non-detected cytokines

IL-2	IL-3	IL-4	IL-2
IL-7	IL-10	IL-12	IL-13
IL-17	IL-23	IL-27	CCL4
CCL11	CCL12	CCL17	CXCL9
CXCL12	GM-CSF	IFN-γ	TNF-α

Supplementary figure 5. Cytokines and inflammatory-associated molecules released within the bone defect. Calvarial defects (4 mm diameter) were treated with fibrin matrices. Fibrin matrices and bone tissue surrounding the defects (1 mm farther) were collected at different time points. Cytokines and inflammatory-associated molecules present within the harvested fibrin matrix/tissue were detected using a protein array. Interleukin (IL), CC chemokine ligand (CCL), chemokine (C-X-C motif) ligand (CXCL), macrophage colony-stimulating factor (M-CSF), granulocyte colony-stimulating factor (G-CSF), soluble intercellular adhesion molecule-1 (sICAM-1), complement component 5 (c5), complement component 5a (C5a), TIMP metallopeptidase inhibitor 1 (TIMP-1), granulocyte-macrophage colony-stimulating factor (GM-CSF), interferon gamma (IFN-γ), tumor necrosis factor alpha (TNF-α). Data are means ± SEM ($n \geq 3$). The list of non-detected cytokines is shown.

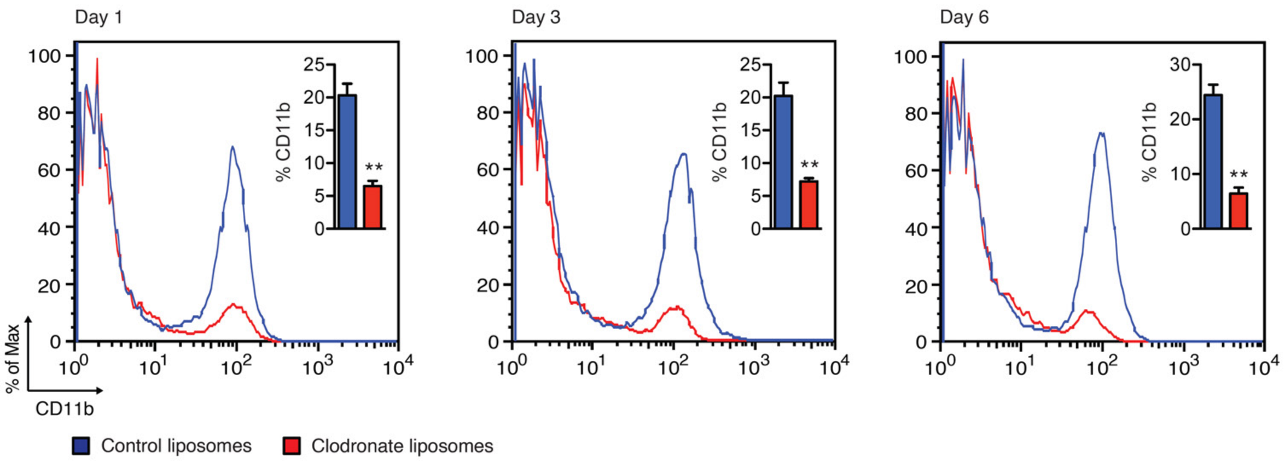

Supplementary figure 6. Depletion of monocytes/macrophages with clodronate liposomes. Clodronate liposomes or control liposomes were intravenously injected in wt mice (200 μl) 2 days prior calvarial surgery. Additional 50 μl of clodronate liposomes or empty liposomes were intravenously injected right before surgery and every 2 days until day 6. The graphs show representative flow cytometry plots and the percentage of $CD11b^+$ collected from the spleen at the indicated days. Data are means ± SEM ($n \geq 3$ per time point). $^{**}P < 0.01$; Student's *t*-test. The majority of monocytes/macrophages were depleted with clodronate liposomes.

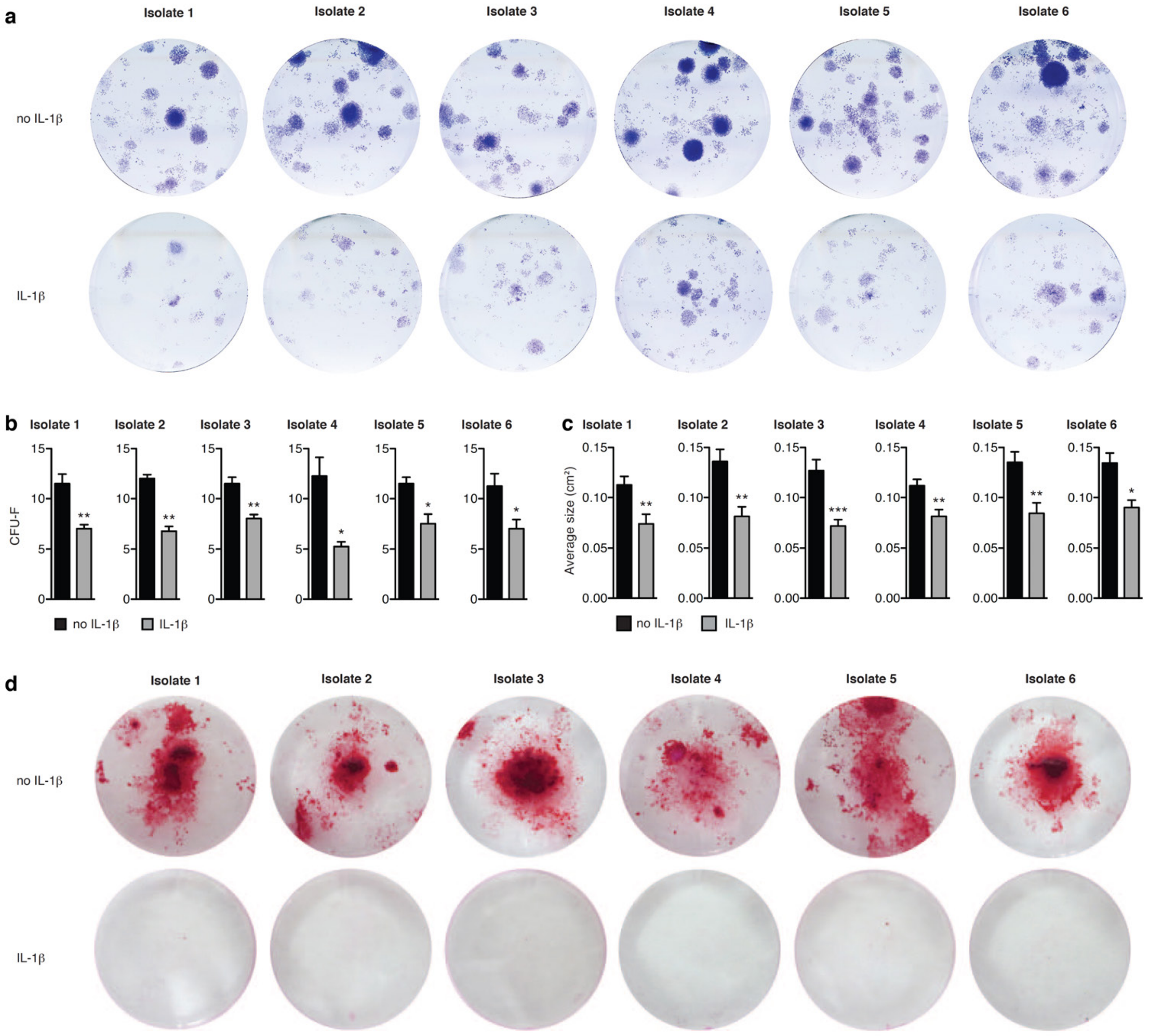

Supplementary figure 7. IL-1β effect on primary CFU-F and differentiation towards osteoblasts of primary bone-derived MSCs. (**a–c**) Colony formation of primary bone-derived MSCs. Cells were seeded with or without IL-1β (1 ng ml^{-1}) and cultured for 12 days. (a) Representative wells (9 cm^2) containing colonies stained with crystal violet. (b) Colony-forming unit-fibroblasts (CFU-F). (c) Average size of colonies. Data are means ± SEM ($n = 4$ per isolate). *$P < 0.05$, **$P < 0.01$, ***$P < 0.01$; Student's *t*-test. (**d**) Cells were seeded in osteogenesis induction medium with or without IL-1β (1 ng ml^{-1}). After 28 days, matrix mineralization was revealed with alizarin red staining. Representative wells (2 cm^2) are shown. Inhibition of colony formation and differentiation into osteoblasts by IL-1β was similar for all isolates.

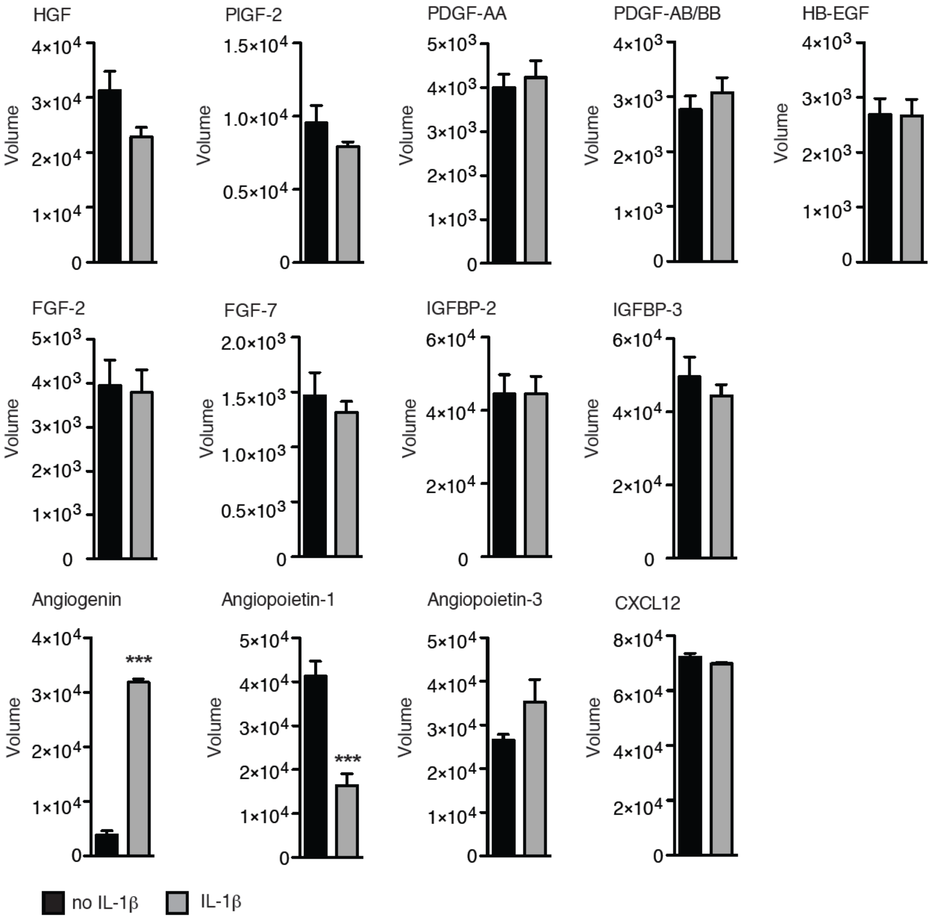

Non-detected trophic factors

Amphiregulin	GM-CSF	IGFBP-10	PD-ECGF
EGF	IGFBP-1	Leptin	VEGF
FGF-1	IGFBP-9	Osteopontin	VEGF-B

Supplementary figure 8. IL-1β effect on MSC trophic factor secretion. MSCs were treated with IL-1β (1 ng ml^{-1}). After 24 h, trophic factors secreted by MSCs were detected using an antibody array. Data are means ± SEM (n = 3). ***$P < 0.001$; Student's *t*-test. Hepatocyte growth factor (HGF), placenta growth factor (PlGF), platelet-derived growth factor (PDGF), heparin-binding epidermal growth factor (HB-EGF), fibroblast growth factor (FGF), insulin-like growth factor binding-protein (IGFBP), chemokine (C-X-C motif) ligand 12 (CXCL12) also known as SDF-1. IL-1β does not significantly influence trophic factor secretion, expect for angiogenin and angiopoietin-1.

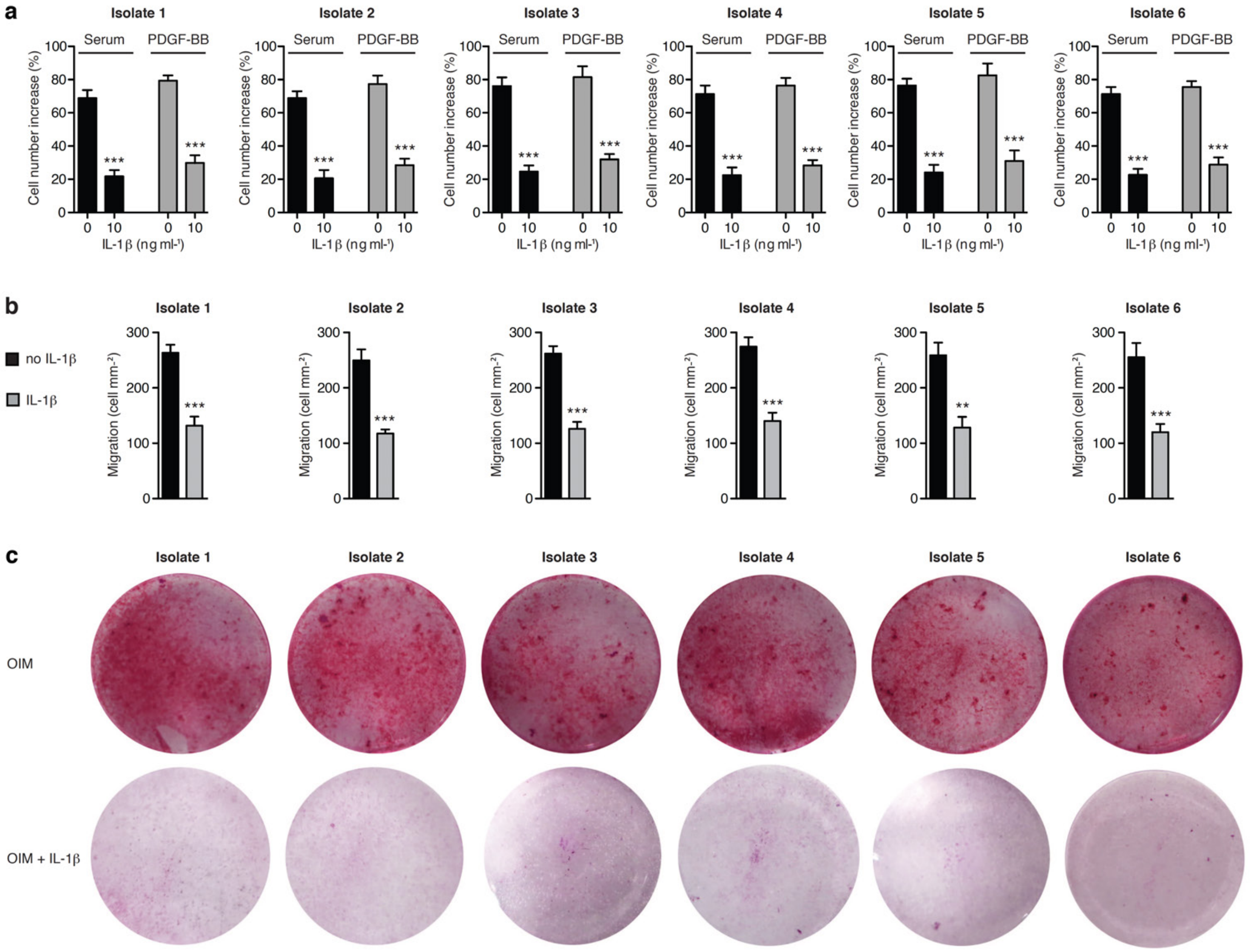

Supplementary figure 9. IL-1β effects on expended MSCs from independent isolates. (**a**) MSCs were stimulated with serum or PDGF-BB (5 ng ml^{-1}), in the presence of IL-1β (5 ng ml^{-1}). Cell number increase was determined after 72 h. (**b**) Migration of MSCs through a migration transwell was induced by PDGF-BB (5 ng ml^{-1}), in the presence of IL-1β (1 ng ml^{-1}). After 6 h, the number of cells per square millimeter that passed through the transwell was counted. For panels a and b, data are means ± SEM (n = 3 per isolate). ***$P <$ 0.01; Student's *t*-test. (**c**) MSCs were cultured in osteogenesis induction medium (OIM) with or without IL-1β (1 ng ml^{-1}). Matrix mineralization was revealed with alizarin red staining, after 28 days of culture. Representative wells (2 cm^2) are shown. The inhibitory effects of IL-1β were similar for all isolates.

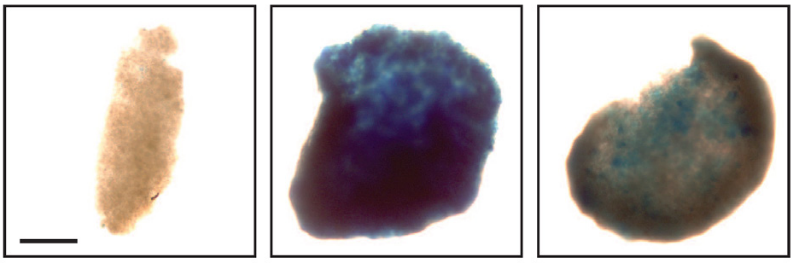

Supplementary figure 10. IL-1β effect on MSC differentiation into chondrocytes. MSCs spheroids in chondrogenesis inducing medium (CIM) were treated once with IL-1β (1 ng ml^{-1} during 4 days). After 28 days, differentiation of MSCs into chondrocytes was revealed by staining glycosaminoglycans (in blue). Representative spheroids are shown. Scale bar = 1 mm. IL-1β inhibits MSC differentiation into chondrocytes.

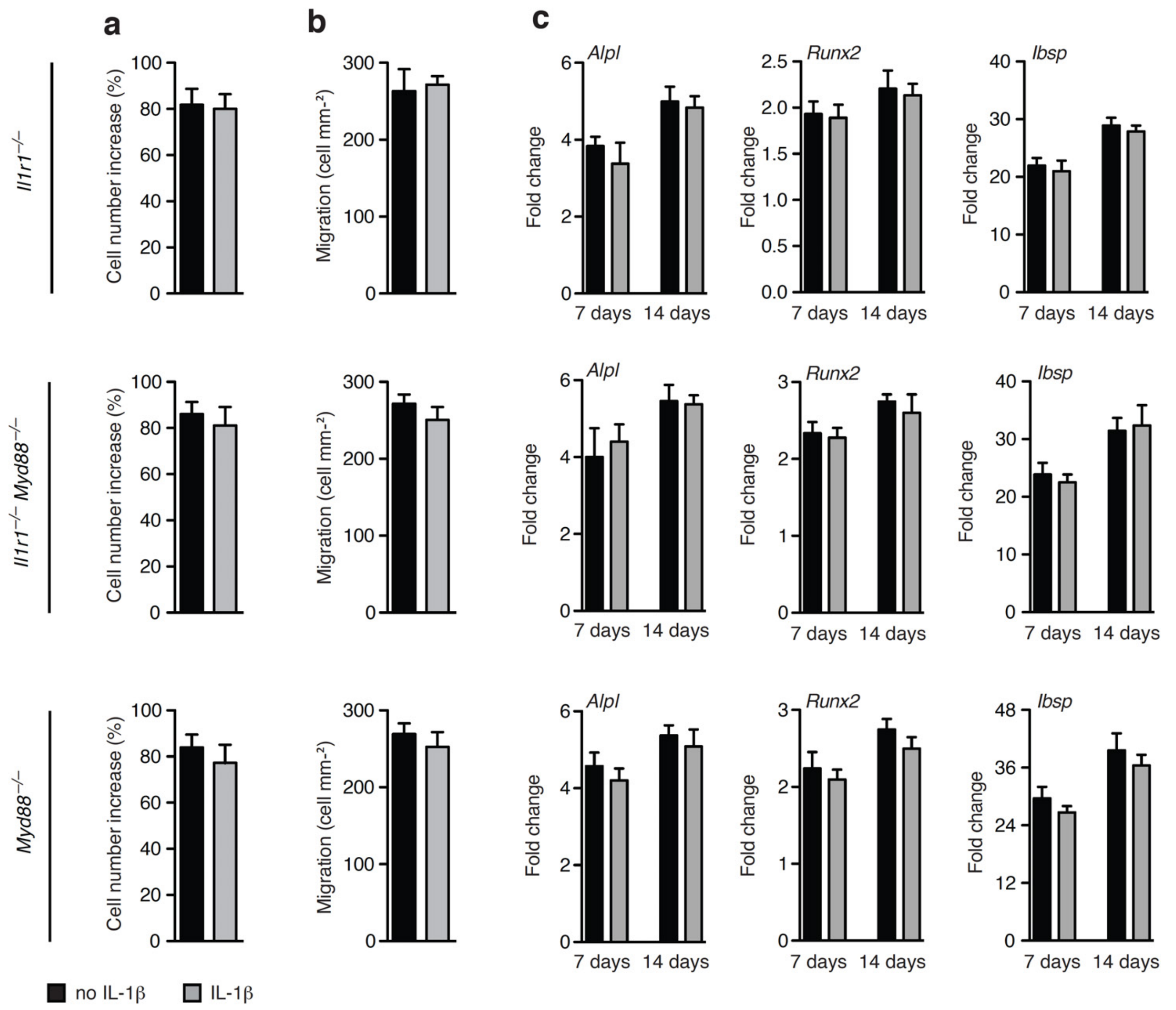

Supplementary figure 11. IL-1β effects on MSCs lacking IL-1R1, MyD88, or both. (**a**) MSCs from *Il1r1*$^{-/-}$, *Myd88*$^{-/-}$, and *Il1r1/Myd88*$^{-/-}$ mice were stimulated with PDGF-BB (5 ng ml^{-1}), in the presence of IL-1β (5 ng ml^{-1}). Cell number increase was determined after 72 h. (**b**) Migration of MSCs through a migration transwell was induced by PDGF-BB (5 ng ml^{-1}), in the presence of IL-1β (1 ng ml^{-1}). After 6 h, the number of cells per square millimeter that passed through the transwell was counted. (**c**) MSCs were cultured in osteogenesis induction medium with or without IL-1β (1 ng ml^{-1}). Expression of osteoblast-specific genes was determined by quantitative PCR. Fold changes in gene expression relative to MSCs cultured in normal medium are shown. *Alpl*, alkaline phosphatase; *Runx2*, runt-related transcription factor 2; *Ibsp,* integrin-binding sialoprotein. For panels a–c, data are means ± SEM (n = 3). No statistical differences were found using Student's t-test. IL-1β has no inhibitory effects on proliferation, migration, and osteoblastic differentiation in MSCs lacking IL-1R1, MyD88, or both.

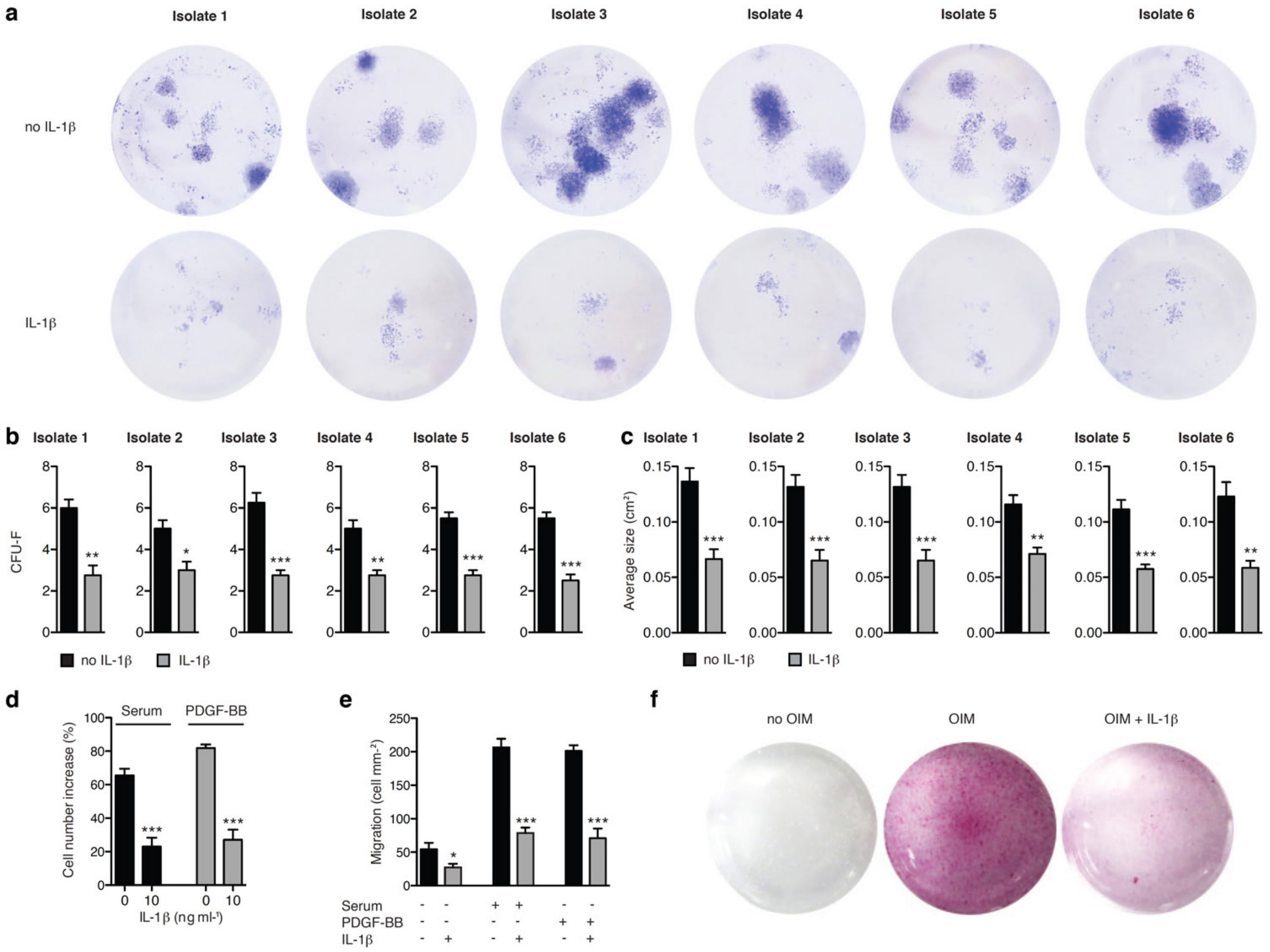

Supplementary figure 12. IL-1β effects on bone marrow-derived MSCs. (**a–c**) Colony formation of primary bone marrow-derived MSCs. Cells were cultured with or without IL-1β (1 ng ml^{-1}) for 12 days. (a) Representative wells (3.5 cm^2) containing colonies stained with crystal violet. (b) Colony-forming unit-fibroblasts (CFU-F). (c) Average size of colonies. Data are means ± SEM ($n = 4$ per isolate). *$P < 0.05$, **$P < 0.01$, ***$P < 0.01$; Student's *t*-test. (**d–f**) IL-1β effect on bone marrow-derived MSCs that have been expended until 3 passages. (**d**) MSCs were stimulated with 10% serum or PDGF-BB (5 ng ml^{-1}), in the presence of 10 ng ml^{-1} of IL-1β. After 72 h, the cell number increase was measured. (**b**). Migration of bone marrow-derived MSCs through a migration transwell was induced with 10% serum or PDGF-BB (5 ng ml^{-1}), in the presence of IL-1β (1 ng ml^{-1}). After 6 h, the number of cells per square millimeter that passed through the transwell was measured. (**c**) Bone marrow-derived MSCs were treated once with IL-1β (1 ng ml^{-1}) and cultured with an osteogenesis induction medium (OIM). After 28 days, matrix mineralization was revealed with alizarin red staining. Representative wells are shown. Similarly to bone-derived MSCs, IL-1β inhibits colony formation, proliferation, migration, and osteoblastic differentiation of bone marrow derived-MSCs.

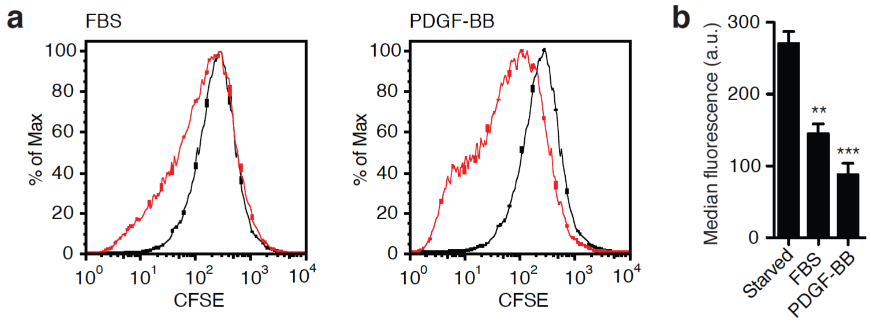

Supplementary figure 13. Proliferation of CFSE-labeled MSCs *in vitro*. CFSE-labeled MSCs from wt mice were stimulated with 1% FBS, 10% FBS, or with 10% FBS plus 5 ng ml^{-1} of PDGF-BB to ensure more than 2 division cycles after 1 week. After 7 days, differences in proliferation were determined by comparing MSC fluorescence intensities using flow cytometry. (**a**) Representative histogram plots are shown. Black curves represent cells cultured with 1% serum (no proliferation control). (**b**) Median fluorescence of MSCs. Data are means ± SEM (n = 3). **$P < 0.01$, ***$P < 0.01$; Student's t-test. Proliferation of CFSE-labeled MSCs can be detected by monitoring their fluorescence, but distinct division cycles were not observed. Because CSFE signal directly depends on cell size, a cell population such as MSCs that has a rather wide size distribution could appear as a wide peak and cell cycle distinction may be indistinguishable. Nevertheless, CSFE-labeling is enough to measure MSC proliferation in a semi-quantitative manner.

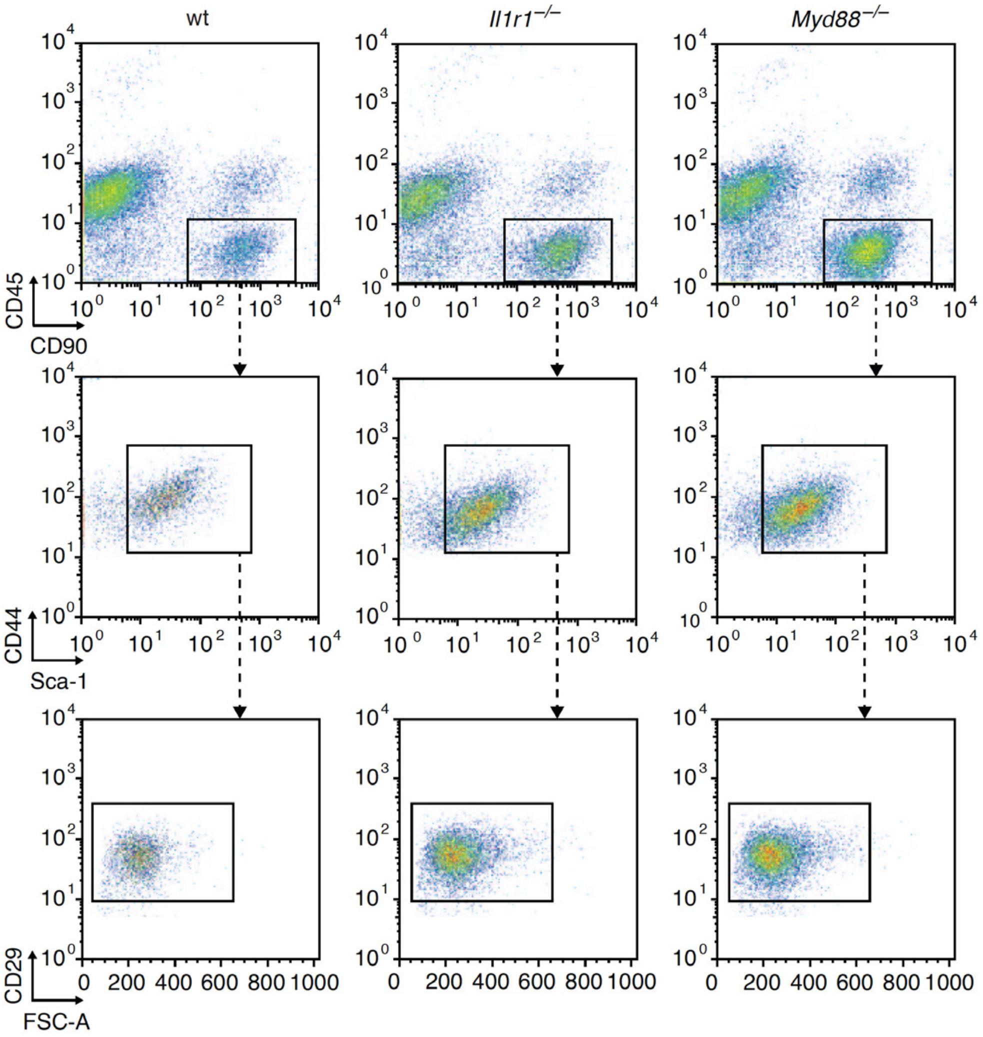

Supplementary figure 14. MSC-like cells mobilization following bone injury. Calvarial defects in wt, *Il1r1*$^{-/-}$, and *Myd88*$^{-/-}$ mice were treated with a fibrin matrix containing PDGF-BB (5 µg) to stimulate stem/progenitor cells mobilization. Seven days after treatment, the percentages of MSC-like cells (CD45^{-}, CD90^{+}, CD44^{+}, Sca-1^{+}, CD29^{+}) recruited within the fibrin matrix were analyzed by flow cytometry. Representative dot plots are shown. More MSC-like cells are mobilized in *Myd88*$^{-/-}$ and *Il1r1*$^{-/-}$ mice compared to wt mice.

	Proteins	Phosphorylation sites
1	Positive control	
2	Negative control	
3	ERK1/2	Thr202/Tyr204
4	Stat1	Tyr701
5	Stat3	Tyr701
6	Akt	Thr308
7	Akt	Ser473
8	AMPKα	Thr172
9	S6RP	Ser235/236
10	mTOR	Ser2448
11	HSP27	Ser78
12	Bad	Ser112
13	p70 S6 Kinase	Thr389
14	PRAS40	Thr246
15	p53	Ser15
16	p38	Thr180/Tyr182
17	JNK	Thr183/Tyr185
18	PARP	Asp214
19	Caspase-3	Asp175 (cleavage)
20	GSK-3β	Ser9

Array map

1	3	3	4	4	1
5	5	6	6	7	7
8	8	9	9	10	10
11	11	12	12	13	13
14	14	15	15	16	16
17	17	18	18	19	19
1	20	20	2	2	2

No stimulation

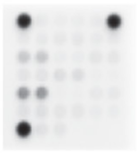

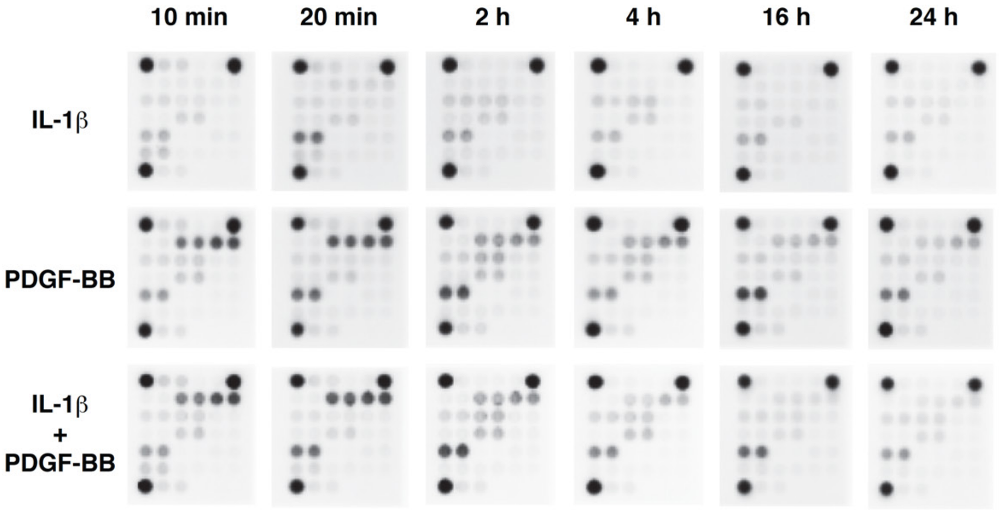

Supplementary figure 15. Signaling of PDGF-BB and IL-1β in MSCs. MSCs were stimulated with PDGF-BB (5 ng ml^{-1}) with or without IL-1β (1 ng ml^{-1}) for 10 min, 20 min, 2 h, 4 h, 16 h, and 24 h. Phosphorylation and cleavage of intracellular singling molecules were analyzed using an antibody array. Representative arrays are shown. Extracellular signal-regulated kinase (ERK), serine/threonine kinase (Akt, also referred to as protein kinase B (PKB)), proline-rich Akt substrate of 40 kDa (PRAS40), ribosomal protein S6 (S6RP), Bcl-2-associated death promoter (Bad), glycogen synthase kinase 3 beta (GSK-3β*),* c-Jun N-terminal kinase (JNK, also referred to as stress-activated kinases (SAPK)), signal transducer and activator of transcription (STAT)*,* 5' AMP-activated protein kinase alpha (AMPKα), mammalian target of rapamycin (mTOR), heat shock protein 27 (HSP27), serine/threonine kinase p70 S6 (p70 S6 Kinase), tumor proteins p53 (p53), p38 mitogen-activated protein kinase (p38), poly ADP ribose polymerase (PARP), cysteine-aspartic acid protease 3 (Casapase-3).

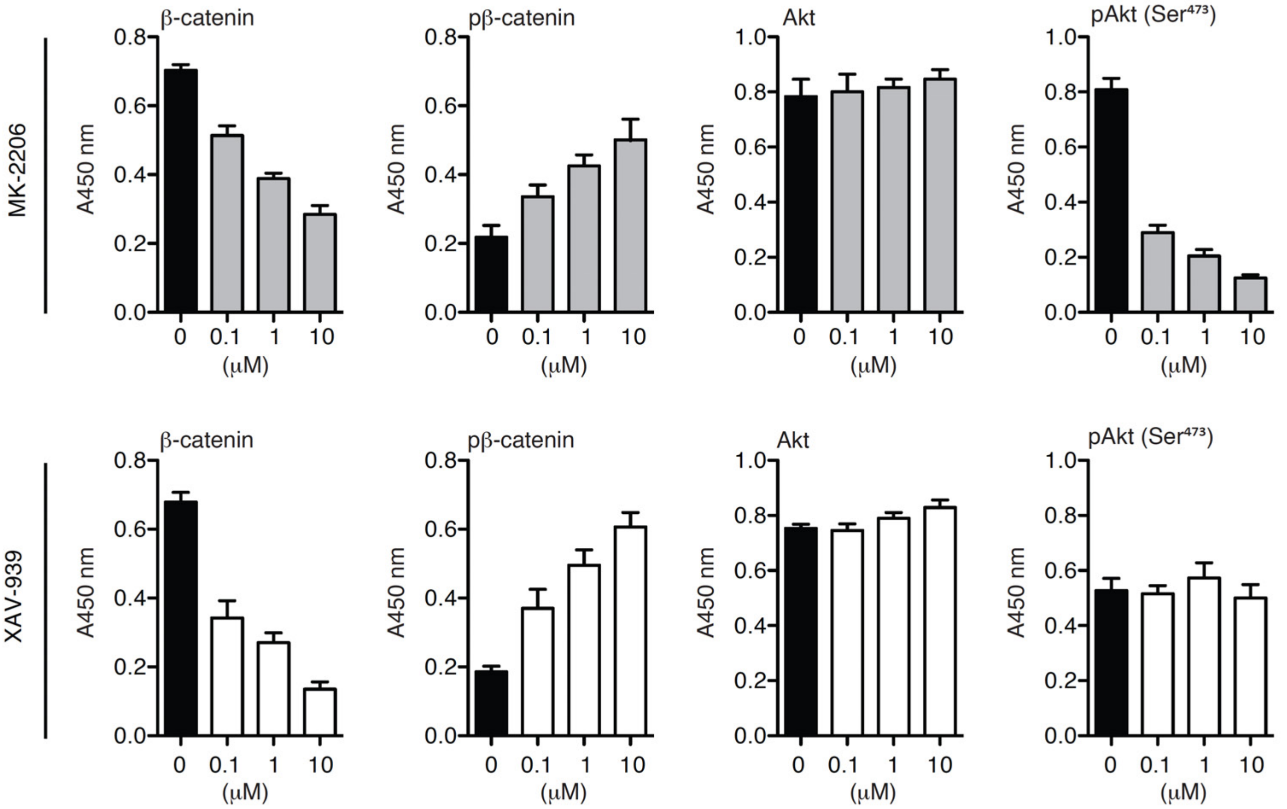

Supplementary figure 16. Efficiency of Akt and β-catenin inhibitors in MSCs. MSCs were stimulated with PDGF-BB (5 ng ml^{-1}) plus MK-2206 or XAV-939 at increasing concentration. After 24h, total Akt, phosphorylated Akt (pAkt), total β-catenin, and phosphorylated β-catenin (pβ-catenin) levels were measured using enzyme-linked immunosorbent assay. Data are means ± SEM (*n* = 3). MK-2206 and XAV-939 are potent inhibitors of Akt and β-catenin, respectively.

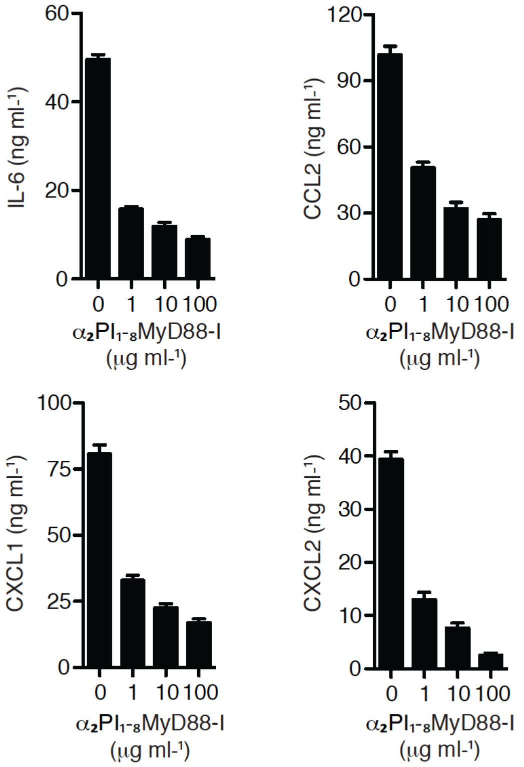

Supplementary figure 17. Inhibitory activity of $\alpha_2PI_{1\text{-}8}$-MyD88-I. MSCs were stimulated with IL-1β (10 ng ml^{-1}) in the presence of $\alpha_2PI_{1\text{-}8}$-MyD88-I at increasing concentration. After 24 h, the concentrations of interleukin-6 (IL-6), chemokine (C-C motif) ligand 2 (CCL2), chemokine (C-X-C motif) ligand 1 (CXCL1), and chemokine (C-X-C motif) ligand 2 (CXCL2) released in the medium were measured by ELISA. Data are means ± SEM (n = 3). The peptide $\alpha_2PI_{1\text{-}8}$-MyD88-I efficiently inhibits IL-1R1/MyD88 signaling.
